# Tracking pyrethroid resistance in arbovirus mosquito vectors: mutations I1532T and F1534C in *Aedes albopictus* across Europe

**DOI:** 10.1186/s13071-025-07130-1

**Published:** 2025-12-24

**Authors:** Verena Pichler, Vera Valadas, Mustafa M. Akiner, Georgios Balatsos, Carlos Barceló, Maria Louise Borg, Jeremy Bouyer, Daniel Bravo-Barriga, Ruben Bueno, Beniamino Caputo, Francisco Collantes, Sarah Delacour-Estrella, Enkelejda Velo, Elena Falcuta, Eleonora Flacio, Ana L. García-Pérez, José F. Gómez, Cintia Horvath, Katja Adam, Perparim Kadriaj, Mihaela Kavran, Gregory L’Ambert, Riccardo P. Lia, Eduardo Marabuto, Raquel Medialdea-Carrera, Rosario Melero-Alcibar, Antonios Michaelakis, Andrei Daniel Mihalca, Martina Micocci, Ognyan Mikov, Miguel A. Miranda, Pie Müller, Concepción Ornosa, Raimundo Outerelo, Domenico Otranto, Igor Pajovic, Javier Pérez-Tris, Dusan Petric, Maria Teresa Rebelo, Gilles Besnard, Elton Rogozi, Ana Tello, Ángeles Vázquez, Marlen Vasquez, Toni Zitko, Francis Schaffner, Alessandra della Torre, Joao Pinto

**Affiliations:** 1 Unicamillus- Saint Camillus International University of Health Sciences, Rome, Italy; 2 Department of Public Health and Infectious Disease, Sapienza University, Rome, Italy; 3 Global Health and Tropical Medicine, GHTM, Associate Laboratory in Translation and Innovation Towards Global Health, LA-REAL, Instituto de Higiene e Medicina Tropical, IHMT, Universidade NOVA de Lisboa, UNL, Rua da Junqueira 100, 1349-008 Lisbon, Portugal; 4 Recep Tayyip Erdoğan Üniversitesi, Rize, Turkey; 5 Laboratory of Insects and Parasites of Medical Importance, Scientific Directorate of Entomology, and Agricultural Zoology, Benaki Phytopathological Institute, Athens, Greece; 6 ZAP-UIB. University of the Balearic Islands, Palma, Spain; 7 Infectious Disease Prevention and Control Unit (IDCU) – Health Promotion and Disease Prevention Directorate, Pietà, Malta; 8 Insect Pest Control Laboratory, Joint FAO/IAEA Centre of Nuclear Techniques in Food and Agriculture, Vienna, Austria; 9 ASTRE, CIRAD, INRAE, University of Montpellier, Plateforme Technologique CYROI, 97491 Sainte Clotilde, France; 10 Department of Animal Health, (Parasitology and Parasitic Diseases), Faculty of Veterinary Medicine, University of Cordoba, UIC Zoonosis and Emerging Diseases (ENZOEM), Sanidad Animal Building, Rabanales Campus, Córdoba, Spain; 11 Laboratorios Lokímica, European Center of Excellence for Vector Control, Rentokil Initial, Spain; 12 Departamento de Zoología y Antropología Física, Universidad de Murcia, Murcia, Spain; 13 University of Zaragoza, Saragossa, Spain; 14 Institute of Public Health, Tirana, Albania; 15 Cantacuzino National Military Medical Institute for Research and Development, Bucharest, Romania; 16 University of Applied Sciences and Arts of Southern Switzerland, Manno, Switzerland; 17 Neiker ‑ Basque Institute for Agricultural Research and Development, Derio, Spain; 18 Department of Biodiversity, Ecology and Evolution. Faculty of Biological Sciences, Complutense University of Madrid, Madrid, Spain; 19 Department of Parasitology and Parasitic Diseases, University of Agricultural Sciences and Veterinary Medicine of Cluj-Napoca, 400372 Cluj-Napoca-Napoca, Romania; 20 University of Primorska, Koper, Slovenia; 21 Faculty of Agriculture, Center of Excellence- One Health, University of Novi Sad, Novi Sad, Serbia; 22 Entente Interdépartementale pour la Démoustication du Littoral Méditérranéen, Montpellier, France; 23 Università degli studi di Bari Aldo Moro, Bari, Italy; 24 Museum of Zoology, Senckenberg Natural History Collections Dresden, Dresden, Germany; 25 National Centre of Infectious and Parasitic Diseases, Sofia, Bulgaria; 26 Swiss Tropical and Public Health Institute, 4123 Allschwil, Switzerland; 27 University of Basel, 4001 Basel, Switzerland; 28 Department of Veterinary Clinical Sciences, City University of Hong Kong, SAR, Hong Kong, China; 29 University of Montenegro, Podgorica, Montenegro; 30 cE3C_CHANGE, Faculdade de Ciências da Universidade de Lisboa, Lisbon, Portugal; 31 Entente Interdépartementale Rhône-Alpes pour la Démoustication, Chindrieux, France; 32 Department of Chemical Engineering, Cyprus University of Technology, Limassol, Cyprus; 33 Institute of Public Health of Split-Dalmatia County, Split, Croatia; 34 BioSys - EI Schaffner Francis, Steinbach, France

**Keywords:** Mosquito, *Aedes albopictus*, Insecticide resistance, *Kdr*, Arbovirus vector, Vector control, Europe

## Abstract

**Background:**

With the worldwide spread of the Asian tiger mosquito, *Aedes albopictus*, the number of autochthonous cases of exotic arboviral diseases, such as dengue or chikungunya, is increasing in temperate regions. In Europe, pyrethroids are the only insecticides allowed for the abatement of adult mosquitoes and are thus crucial for limiting ongoing arbovirus transmission. Despite this and the report of resistance rising in vector populations worldwide, information on the pyrethroid resistance status of vector populations and knowledge on resistance mechanisms is widely lacking. Genotyping of knockdown resistance (*kdr*) mutations situated within the target site of pyrethroids, i.e., the voltage-gated sodium channel (VGSC), and associated with pyrethroid resistance, is a cost-effective approach to investigate the spread of resistance in a population. Herein, we describe the European-wide distribution of two *kdr* mutations, i.e., I1532T and F1534C, in *Ae. albopictus* and evaluate their co-occurrence with another well-characterized *kdr* mutation, V1016G.

**Methods:**

Genotyping of the *kdr* mutation F1534C was performed by allele-specific PCR for 1732 *Ae. albopictus* specimens sampled in 19 European countries; for a subset of 419 specimens mutation I1532T was also genotyped by sequencing. For all samples, information on mutation V1016G was available, allowing evaluation of the co-occurrence of *kdr* alleles.

**Results:**

Mutation 1534C was detected in nine sites from six countries at an overall frequency close to 5%. Highest frequencies per site were detected in Cyprus (84%) and Greece (45%). Allele 1532 T was identified in 11 sites from 7 countries at frequencies ranging from 4% to 25% per site. Co-occurrence of different *kdr* alleles (1534C, 1532 T and 1016G) was observed in nine sampling sites from seven countries.

**Conclusions:**

The present study offers the first map of the occurrence of the major *Ae. albopictus kdr* alleles across Europe and highlights a differential distribution of the two alleles most strongly associated with pyrethroid resistance, 1016G and 1534C. Our findings also point to the need for enhancing resistance monitoring in the Eastern Mediterranean region, where the two mutations are shown to exist in geographically close areas, with the risk of emergence of highly resistant double mutants.

**Graphical Abstract:**

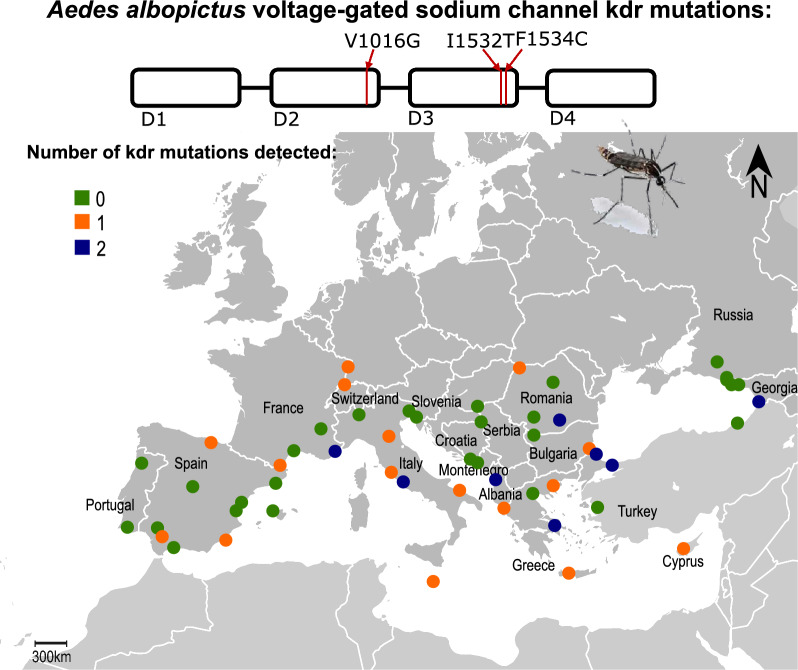

**Supplementary Information:**

The online version contains supplementary material available at 10.1186/s13071-025-07130-1.

## Background

The last few decades have seen an important re-emergence of mosquito-borne diseases. Dengue cases have increased ten times over the last 20 years [1, 2], and Zika virus spread rapidly across the globe in 2015–2016 [3]. Diseases previously thought to be restricted mainly to tropical areas are becoming more common in temperate regions, including Europe.

The Asian tiger mosquito *Aedes albopictus* (Skuse, 1894), native to Southeast Asia, has extensively expanded its distribution during the last few decades, invading most parts of central and southern Europe [4–7]. This is mainly owing to the species’ ability to form diapausing eggs, which can resist cold winters in temperate regions and facilitate transport over long distances. The ongoing processes of globalization and climate change are expected to further favour the species’ range expansion [8]. The increasing presence of *Ae. albopictus* in Europe is a growing concern, given its ability to act as a vector of several arboviruses, the most important ones being chikungunya (CHKV) and dengue (DNV). Indeed, *Ae. albopictus* was responsible for two CHKV outbreaks in 2007 and 2017 in Italy, with more than 700 notified cases [9, 10], and for an increasing number of autochthonous dengue cases in Mediterranean Europe, with more than 300 locally acquired cases in Italy, France and Spain in 2024 [11–13].

In Europe, pyrethroid insecticides are the only chemicals officially authorized to be used for emergency interventions to rapidly reduce *Ae. albopictus* abundance and limit arbovirus transmission in case of detection of infected human cases [14–16]. However, despite the European Centre for Disease Prevention and Control (ECDC) recommending focusing on larval control as a preventive measure, pyrethroids are also frequently used to prevent mosquito nuisance. In addition, pyrethroids are widely used in agriculture, contributing to the overall selective pressure for resistance in mosquito populations [17]. This extensive use raises environmental and public health concerns and promotes the emergence of insecticide resistance [18].

In mosquitoes, resistance mechanisms are mainly based on the overexpression of detoxification enzymes or on mutations at the insecticides’ target-site [19]. Pyrethroids bind to the voltage-gated sodium channel (VGSC), which is involved in nervous signal transmission, and interfere with its correct action, leading to knockdown and death of the mosquito. Several amino acid substitutions – commonly referred to as knockdown resistance (*kdr*) mutations – interfere with pyrethroid binding, eventually lowering their effectiveness. Different *kdr* mutations have been identified in mosquitoes and are known to be widespread in main tropical vectors of malaria and arboviruses, such as *Anopheles gambiae* and *Aedes aegypti*, respectively [20–23]. However, information on these mutations in temperate-region vectors including *Ae. albopictus* remains limited. In this species, the strongest support for an association with a pyrethroid resistance (PR) phenotype exists for mutations at positions 1016 and 1534 of the VGSC [24–26]. These two mutations are situated in segment S6 of the transmembrane domains II and III, respectively, and are involved in altering the pyrethroid receptor site 1 [21].

Substitution F1534C was the first *kdr* mutation to be identified in *Ae. albopictus*, first in specimens from Singapore [27] and subsequently in populations from several countries around the world [25, 26, 28]. The discovery of other allelic variants for the same amino acid position, i.e., 1534 S/L/R/W, as well as in the nearby position 1532, further stressed the selective pressure on this genomic region [26, 29]. Substitution V1016G was identified subsequently by Kasai et al. [24] in populations from Vietnam and Italy, and since then it has been reported in several European and Asian countries [26, 30].

Several studies investigating resistance phenotypes associated with these mutations have been performed [24, 26, 29, 31–33], often with conflicting outcomes, suggesting that metabolic resistance mechanisms as well as differences in the genetic background strongly impact the phenotype. Kasai et al. [24] tried to avoid such biases by investigating mosquito colonies carrying the same genetic background but different *kdr* mutations and exposed them to different pyrethroids in combination with PBO, thus avoiding the bias introduced by the possible presence of metabolic mechanisms. This approach allowed us to confirm the role of alleles 1016G, 1534C and 1534S in conferring resistance to different classes of pyrethroids (type I and type II). Less information exists for the nearby mutation I1532T, but functional studies performed by Yan et al. [34] suggested a role of this mutation in conferring resistance to type I pyrethroids. Notably, mutation F1534C is found also in *Ae. aegypti* populations across all continents except Australia [23, 25] and has been shown to confer resistance both alone and in combination with other *kdr* mutations, such as V1016G/I [23, 25, 26, 35].

The resistance status of mosquito populations is usually assessed by the implementation of standardized bioassays [36]. In Europe, susceptibility to different pyrethroids has been investigated in populations from Albania, Greece, Italy and Spain [32, 37–42], and PR was recorded in populations from Italy and Spain [30, 43]. However, these assays are time-consuming and require working with large numbers of live mosquitoes, often hindering large-scale investigations. However, once the association of a given mutation with a PR phenotype is established, investigating the presence of this mutation in mosquito populations is a valuable indicator of resistance [36]. Genotyping assays are indeed easy and cost-effective and can be performed in most molecular biology laboratories on large numbers of specimens regardless of sex and developmental stage. Moreover this approach allows us to detect the circulation of resistance alleles at low frequencies and before having any impact on the resistance phenotype, which is useful for implementing timely PR management strategies.

In Europe, only the distribution of the V1016G mutation has been investigated thus far, highlighting the widespread presence of this mutation across Italy, where it reaches frequencies above 40% per site, and low frequencies in eight other countries [44, 45]. Conversely, little information is available concerning the presence of other *kdr* alleles. Mutations in positions 1532 and 1534 have been investigated mainly in Albania, Greece and Italy, with allele 1532 T being detected in all three countries and allele 1534C in Greece [32, 37, 46].

Thanks to the extensive collaboration of European medical entomologists within the framework of two large projects (ARBOMONITOR and the AIM-COST Action on *Aedes* invasive mosquitoes), we conducted the first European-wide study on *kdr* mutations F1534C and I1532T in *Ae. albopictus*. The results, in association with previously published data on mutation V1016G, provide a baseline dataset on target site resistance in this species across Europe and identify hotspots where enhanced PR monitoring and management are advisable to prevent failure of pyrethroid-based emergency interventions.

## Methods

### Mosquito collections

Field collections of *Ae. albopictus* eggs, larvae or adults were carried out from August 2015 until October 2022 in 54 municipalities across 19 European countries (Abkhasia, Albania, Bulgaria, Croatia, Cyprus, France, Georgia, Greece, Italy, Malta, Montenegro, Portugal, Romania, Russia, Serbia, Slovenia, Spain, Switzerland and Turkey) (Additional file 1: Supplementary Table S1). Specimens were sent to the Instituto de Higiene e Medicina Tropical at Universidade Nova de Lisboa (Portugal) or to the Department of Public Health and Infectious Diseases of Sapienza University of Rome (Italy) for molecular genotyping.

### DNA extraction

Genomic DNA was extracted from single specimens, using different manual extraction methods [47, 48] or DNA extraction kits (NZY Tissue gDNA Isolation kit—Nzytech, Portugal; DNeasy Blood and Tissue, Qiagen, USA). Extracted DNA was stored at −20 °C until further analysis.

### Genotyping

Genotyping of the F1534C mutation was performed following the AS-PCR protocol described by Zhu et al. [49] using universal primers Zhu_AF and Zhu_AR in combination with allele-specific primers zhu1534F and zhu1534C, able to bind to the wild-type allele 1534F and the *kdr* allele 1534C, respectively. Because the AS-PCR approach is more cost-effective, it was preferred over Sanger sequencing given the large number of specimens to be analysed. To validate F1534C genotypes and to assess the possible presence of mutation I1532T, a 264 bp long fragment of domain III of the *vgsc* gene – comprising partial exon 28, the full intron 28, and partial exon 29 – was sequenced in a subsample that included all specimens identified by AS-PCR as homo- or heterozygotes for allele 1534C, plus randomly selected specimens carrying the wild-type allele 1534F in homozygosis. Amplicons were purified using the Exo/SAP Go—PCR Purification Kit (GRISP, Portugal) according to the manufacturer's protocol and sent for Sanger sequencing at STABVida, Portugal (Oeiras, Portugal), or Eurofins Genomics (Ebersberg, Germany). Sequences were read using the software Chromas version 2.6.6 (Technelysium–2025, Brisbane, Australia) and aligned using MEGA X [50]. Information on the V1016G was retrieved from a previously published study [45], with the exception of the population from Cyprus, which was PCR genotyped for the present study using the PCR approach described by Pichler et al. [44].

### Data analysis

Accuracy of the AS-PCR technique was estimated by dividing the number of correct assessments by the total number of observations, considering the DNA sequencing results as the gold standard. Sensitivity and specificity were computed to evaluate the ability of the AS-PCR to detect the presence or absence of allele 1534C. Sensitivity was computed as the number of true positive assessments as detected by AS-PCR divided by the number of all positive assessments as detected by sequencing. Specificity was computed as the number of true negative assessments in AS-PCR divided by the number of all negative assessments as detected by sequencing. Hardy–Weinberg equilibrium of *kdr* alleles in populations was tested using the chi-square statistical test.

## Results

A total of 1732 *Ae. albopictus* specimens were PCR-genotyped for the F1534C mutation. Of these, 429 specimens were sequenced for the above-described fragment of domain III of the *vgsc* gene. Sequencing allowed to detect three different alleles in position 1534, i.e., the wild-type allele 1534F (encoded by two different triplets, TTC and TTT), and the two *kdr* alleles 1534C (encoded by TGC) and 1534L (encoded by TTG), as well as two alleles in position 1532, i.e., the wild-type allele 1532I (encoded by ATC) and the *kdr* allele 1532 T (encoded by ACC).

### Accuracy of AS-PCR genotyping

Partial sequencing of domain III of the *vgsc* gene was successful for 419 out of 429 specimens with sequences available in Additional file 2. Overall concordance between AS-PCR and sequencing was 93% (Table 1) with a sensitivity for the detection of the 1534C allele (at the homozygous or heterozygous state) of 95% and a specificity of 94%. Discordant results were identified in 29 specimens. In 20 specimens (71%) these were due to a heterozygote result in AS-PCR identified as 1534F/1534F homozygotes by sequencing. Two of the specimens were heterozygotes for two different codons (TTC/TTT) encoding allele 1534F, explaining the genotyping error. For the remaining 18 specimens, nonspecific binding of the 1534C-specific primer, resulting in an overestimation of the 1534C allele frequency, is the most plausible hypothesis. Three 1534C/1534F heterozygotes by AS-PCR were found to be 1534C/1534C homozygotes after sequencing. Five specimens were identified as 1534F/1534F homozygotes by AS-PCR but were either 1534C/1534F heterozygotes (*N* = 3) or 1534C/1534C homozygotes (*N* = 2) after sequencing. Especially in the latter case, human error cannot be ruled out. Finally, a single specimen from Italy genotyped as a wild-type 1534F homozygote by AS-PCR was identified by sequencing as a heterozygote for the wild-type allele together with allele 1534L, impossible to detect with this AS-PCR approach.

**Table 1:** Comparison of AS-PCR genotyping and sequencing results for mutation F1534C within the *vgsc* gene in *Aedes albopictus* from Europe. Concordant results are in bold

Sequencing						
		CC	FC	FF	FL	Total
AS-PCR	CC	**38**	–	–	–	38
	FC	3	**51**	20	–	74
	FF	2	3	**301**	1	307
	Total	43	54	321	1	419

*F* allele 1534F, *C* allele 1534C, *L* allele 1534L

### Genotype frequency and distribution of *kdr* alleles

**Fig. 1:**
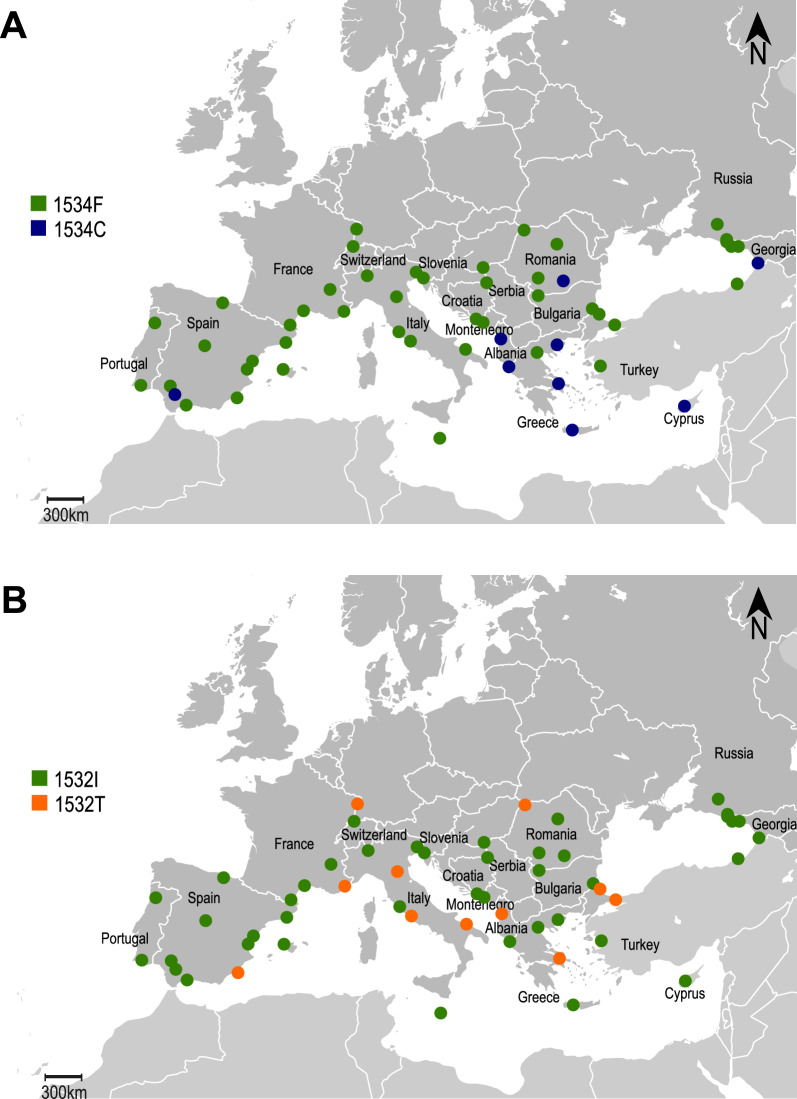
Distribution of *kdr* mutations F1534C (**A**) and I1532T (**B**) in *Aedes albopictus* populations sampled across Europe. Each dot represents a sampling site for which details are available in Additional File 1: Supplementary Table S1. Green dots represent samples where only the respective wild-type alleles (1534F or 1532I) were detected; blue dots represent samples where *kdr* allele 1534C was detected, orange dots where *kdr* allele 1532 T was detected. Allele 1534L, observed only in one specimen from Rome, Italy, in heterozygosis, is not represented

Results of the genotyping of 1732 *Ae. albopictus* specimens from 54 sites in 19 European countries are shown in Table 2, Fig. 1, and in Additional File 1: Supplementary Table S2. For the 29 individuals with conflicting results between AS-PCR and sequencing, the genotype obtained by sequencing was considered for further analysis. Overall, 1623 homozygotes for the wild-type allele 1534F (1534F/1534F), 60 heterozygotes (1534F/1534C), 48 homozygotes for the *kdr* allele 1534C (1534C/1534C) and one 1534F/1534L heterozygote were detected.

**Table 2:** Allele frequencies (AF) per country and number of *Ae albopictus* specimens (N inds) analysed for mutations F1534C and I1532T, along with number of sites (NS) sampled per country

		F1534C AF				I1532T AF		
Country	NS	N Inds	F	C	L	N Inds	I	T
Abkhazia	2	47	1.000	–	–	8	1.000	–
Albania	2	65	0.977	0.023	–	30	0.967	0.033
Bulgaria	2	88	1.000	–	–	13	1.000	–
Croatia	1	46	1.000	–	–	13	1.000	–
Cyprus	1	53	0.160	0.840	–	43	1.000	–
France	5	131	1.000	–	–	18	0.889	0.111
Georgia	1	49	0.949	0.051	–	3	1.000	–
Greece	4	158	0.820	0.180	–	90	0.956	0.044
Italy	5	143	0.997	–	0.003	49	0.898	0.102
Malta	1	50	1.000	–	–	9	1.000	–
Montenegro	1	32	1.000	–	–	15	1.000	–
Portugal	2	76	1.000	–	–	10	1.000	–
Romania	4	108	0.995	0.005	–	12	0.958	0.042
Russia	4	50	1.000	–	–	6	1.000	–
Serbia	2	88	1.000	–	–	11	1.000	–
Slovenia	1	40	1.000	–	–	9	1.000	–
Spain	10	289	0.998	0.002	–	46	0.978	0.022
Switzerland	2	31	1.000	–	–	9	1.000	–
Turkey	4	188	1.000	–	–	25	0.940	0.060
Total	54	1732	0.955	0.045	0.000	419	0.964	0.036

*F* allele 1534F, *C* allele 1534C, *L* allele 1534L; I allele 1532I, *T* allele 1532 T

The 1534C *kdr* allele was found in nine sites from six countries (Albania, Cyprus, Georgia, Greece, Romania and Spain) at frequencies ranging from 1.5% to 84% (Additional File 1: Supplementary Table S2). Almost all (92.6%) of the specimens carrying the 1534C allele in hetero- or homozygosity were detected in Cyprus (*N* = 53) and Greece (*N* = 47). The highest 1534C frequency (84%) was detected in Cyprus (Limassol), where 68% of the specimens carried the 1534C allele in homozygosity and no wild-type homozygotes were found. In Greece, allele 1534C was detected in three out of four sampling sites at frequencies of 10% (Kavala), 12.5% (Chania) and 45% (Athens), with homozygotes being present in Athens (20.8%) and Chania (2.3%). Elsewhere, the 1534C allele was detected in eight specimens from five sites in Albania, Georgia, Romania and Spain, two of which were homozygotes (from Batumi, Georgia). Sequencing allowed the detection of one 1534L/1534F heterozygote in Rome (Italy). The chi-square statistical test found no significant deviation from Hardy–Weinberg equilibrium for genotypes at locus 1534 in any of the sampling sites.

Sequencing results also allowed us to assess the presence of mutation I1532T (Table 2 and Additional File 1: Supplementary Table S2). Overall, 26 specimens from 11 sites in 7 countries (Albania, France, Greece, Italy, Romania, Spain and Turkey) carried the 1532 T allele, 4 of which (sampled in Spain, Italy and Greece) were homozygotes. Frequencies per site ranged from 3.8% (Istanbul, Turkey) to 25% (Italy: Bologna, Romania: Satu Mare). At two locations (Greece: Athens, Albania: Durres), both mutations (F1534C and I1532T) were detected. Moreover, four Greek specimens were identified as carrying both mutated alleles: three were heterozygous for both mutations, and one was heterozygous at position 1532 (1532I/1532 T) and homozygous for the 1534C allele (Additional File 1: Supplementary Table S2).

**Fig. 2:**
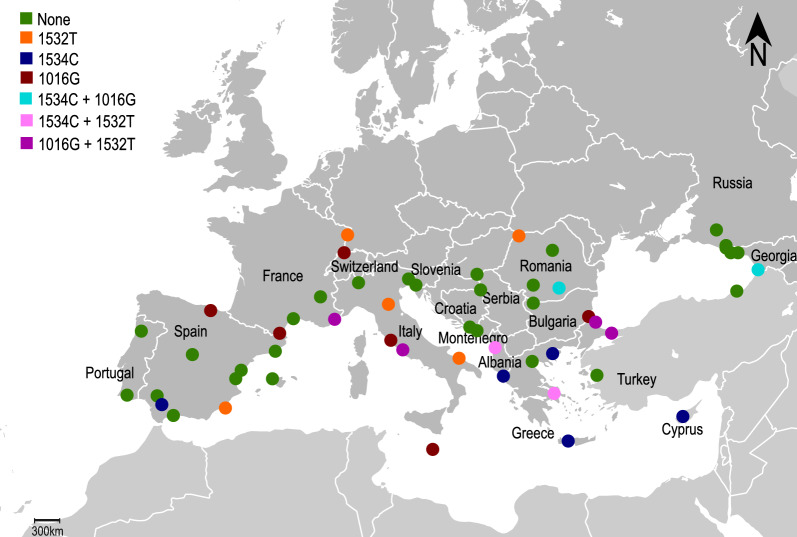
Distribution of *kdr* alleles 1016G, 1532 T and 1534C in *Aedes albopictus* populations across Europe. Data obtained within the present study were merged with data available from Pichler et al.  [45]. Green dots indicate sites where no *kdr* mutation was detected, dark red dots indicate presence of allele 1016G, dark blue dots presence of allele 1534C and orange dots presence of allele 1532 T. Presence of two different *kdr* alleles in the same sampling site is shown by cyan dots (1534C + 1016G), purple dots (1532 T + 1016G) and pink dots (1534C + 1532 T)

For 1671 of the specimens genotyped for mutation F1534C, data on mutation V1016G were available from Pichler et al. [45] and are shown in Fig. 2 and Additional File 1: Supplementary Table S2. For the population from Cyprus, no previous information was available, and thus genotyping was performed for the present study, revealing the absence of the *kdr* allele 1016G in the 53 examined specimens (Additional File 1: Supplementary Table S2). In two populations (Batumi, Georgia and Bucharest, Romania), both *kdr* alleles, 1016G and 1534C, were detected at frequencies below 6%, with one specimen from Batumi being a heterozygote for allele 1016G and a homozygote for allele 1534C. Co-occurrence of alleles 1532 T and 1016G was detected in three countries (Turkey, Italy and France], with one specimen from Rome (wild-type in position 1534) carrying both alleles in heterozygosity.

## Discussion

Despite the spread of pyrethroid resistance threatening our ability to quickly and effectively interrupt the transmission of exotic arboviruses by *Ae. albopictus*, little is known on the magnitude of this problem in Europe. Herein, we map for the first time the distribution across Europe of one of the most well-known *kdr* mutations in *Aedes* mosquitos, F1534C, as well as the adjacent mutation I1532T. Both *kdr* alleles appear to be overall rare (with an overall allelic frequency below 5%), but with important local peaks in frequency for allele 1534C.

Reliable genotyping results were obtained by AS-PCR (i.e. 93% accuracy), with most of the errors observed in homozygous specimens erroneously genotyped as heterozygotes by AS-PCR. Such errors are common in AS-PCR protocols, possibly owing to non-specific binding of one of the two allele-specific primers. In the present study these errors would have led to a slight overestimation of the frequency of the resistance-associated allele. While future research is needed to develop genotyping approaches less prone to this error, at present it is recommended to sequence at least a subset of specimens harbouring the 1534C allele for confirmation of the AS-PCR genotype. Sequencing of a subset of specimens is also recommended owing to the presence of other 1534 allelic variants [26]. Our study suggests that in Europe the most frequent codons at position 1534 are TTC (encoding for the wild-type allele) and TGC (encoding for the *kdr* allele 1534C). Notably, in the *Ae. albopictus* native range, codons TGT and TCC for allele 1534C have also been detected [26, 51].

The present study suggests a hotspot of allele 1534C in the Eastern Mediterranean region with concerningly high frequencies in Cyprus and Greece, while in the rest of Europe the allele appears to be rare. Previous reports for this mutation across Europe are scarce. In Greece, allele 1534C was shown to be widespread in previous studies [32, 37, 42, 52, 53]. Balaska et al. [32] found the 1534C allele in all 12 sites sampled across Greece at frequencies ranging from 6% to 68%. Similar to our results, highest frequencies were detected around Athens (Attika region) and lowest in the area around Thessaloniki. Elsewhere in Europe, the 1534C allele has only been investigated in Italy, where it was not detected before [29, 37]. However, the 1534L allele was observed on one occasion in a northern Italian population (Arco; AF = 1%; [51]). No previous information on populations from Cyprus was available since this island was colonized only recently by *Ae. albopictus* [54, 55]. Interestingly, we observed the isolated presence of the 1534C allele in one site in Spain. Corley et al. [56] observed a similar pattern when analysing *Ae. albopictus* microsatellites, with specimens from one Turkish and one Spanish site showing common ancestries and suggesting a possible origin of Spanish populations from East European ones, which is compatible with the present data.

Allele 1532 T was detected in Albania, Greece and Italy, where it was already reported at frequencies up to 32% [24, 29, 32, 37, 42], as well as for the first time in France, Romania, Spain and Turkey. Overall, the allele appears to be rare in Europe (overall allele frequency below 5%), but frequencies above 10% were detected in Italy and France. However, since this allele can only be identified by sequencing, the number of specimens analysed per site was generally low, thus affecting the possibility of accurately inferring its actual distribution.

Almost all the specimens herein analysed for the presence of mutations at 1534 position had also been genotyped for the presence of *kdr* allele 1016G by Pichler et al. [45]. Interestingly, the two alleles seem to have a generally different distribution in Europe, forming two hotspots across the Mediterranean region, with allele 1534C being more prevalent in Eastern Europe, mainly in Greece and Cyprus, and allele 1016G having the highest frequency in Italy [44, 45]. This could reflect the different origins of the populations in these two geographical regions. Previous studies suggested different *Ae. albopictus* invasion histories for Greece and Italy, with the latter being more closely linked to populations from Japan/USA and the Greek populations clustering instead with populations from Southeast Asia [56–58]. In Cyprus, instead, *Ae. albopictus* was recorded for the first time only in 2022 [54, 55]. A recent study showed that the Cyprus populations shared a high degree of ancestry with those in the Balkans and parts of northern Italy that border the Adriatic Sea [59]. Our results seem to suggest a link of the population from Cyprus with the Greek ones, compatible also with the numerous maritime connections between these two geographical regions.

Another possible explanation for the different distribution of the two alleles might be differences in insecticide usage (e.g., a differential usage of type I and type II pyrethroids), with selective pressure favouring the emergence of distinct *kdr* alleles in the two geographic regions. However, information on both insecticide usage across Europe and possible differences in the resistance phenotype of the two mutations, 1016G and 1534C, is still scarce.

Finally, we found co-occurrence of both alleles, 1016G and 1534C, in two sites, i.e., Batumi (Georgia) and Bucharest (Romania). In these two populations, both *kdr* alleles were found at low frequencies (< 6%), with one specimen from Batumi carrying both alleles. Furthermore, one specimen from Rome was found to be heterozygous for alleles 1532 T and 1016G (and wild-type for position 1534). These few reports of co-occurrence of different *kdr* alleles are relevant, as the possible synergism between mutations may increase the resistant phenotype. Indeed, Hirata et al. [35] showed that an *Ae. aegypti vgsc* haplotype carrying the three mutations S989P + V1016G + F1534C resulted in a 1100-fold reduction in sensitivity to permethrin compared with a 25-fold reduction in sensitivity of F1534C mutants. Moreover, a strong additive effect was observed in double heterozygote *Ae. aegypti* field specimens (V/G1016 + F/C1534) from Malaysia [60, 61] and Thailand [61, 62]. Support for a possible synergism involving the 1532 T allele is still lacking [34]. Overall, the possible synergism between *kdr* alleles should be further investigated and monitored in *Ae. albopictus* across Europe, where the high frequency of alleles 1016G and 1534C in two highly connected geographical regions such as Italy and Greece raises concern.

## Conclusions

The present study represents the first effort to map the combined distribution of different *kdr* alleles (1532 T, 1534C and 1016G) in *Ae. albopictus* across Europe and serves as a baseline for future surveillance and research. The findings reveal distinct geographic patterns for the two most well-characterized *kdr*-alleles, 1016G and 1534C, across the Mediterranean region, with allele 1016G reaching highest frequencies in Italy and 1534C in Cyprus and Greece. Given the dense maritime connections between Italy and Greece, the emergence of recombinant haplotypes carrying both mutations is plausible and may result in enhanced resistance phenotypes. To prevent the establishment and spread of such potentially super-resistant populations, continuous and targeted monitoring of resistance alleles should be prioritized in the Eastern Mediterranean region. Future research should also explore the phenotypic impact and fitness costs of individual mutations, as well as potential synergistic effects among them. This information is crucial for guiding effective vector control strategies and for mitigating the growing public health threat posed by *Ae. albopictus* in Europe.

## Supplementary Information

Additional file 2.

Additional file 1.

## Data Availability

All data is available within the paper and its additional files and representative sequences for alleles at position 1534C and 1532 of the VGSC are available at GenBank accession numbers PX438780-PX438782.

## Abbreviations

*kdr*: Knock-down resistance
VGSC: Voltage-gated sodium channel
PR: Pyrethroid resistance
AS-PCR: Allele-specific polymerase chain reaction
