# Supplementary material for: Tracking pyrethroid resistance in arbovirus mosquito vectors: mutations I1532T and F1534C in Aedes albopictus across Europe
Source: Parasit Vectors. 2025 Dec 24;18:506. doi: 10.1186/s13071-025-07130-1 (PMC12728990; doi:10.1186/s13071-025-07130-1)
Supplement: Supplementary file 1 — Additional file 2. [file 13071_2025_7130_MOESM1_ESM.docx]

**Tracking Pyrethroid Resistance in arbovirus mosquito vectors: Mutations I1532T and F1534C in Aedes albopictus across Europe**

Verena Pichler, Vera Valadas, Mustafa M. Akiner, Georgios Balatsos, Carlos Barceló, Maria Louise Borg, Jeremy Bouyer, Daniel Bravo Barriga, Ruben Bueno, Beniamino Caputo, Francisco Collantes, Sarah Delacour Estrella, Enkelejda Velo, Elena Falcuta, Eleonora Flacio, Ana L. García-Pérez, José F. Gómez , Cintia Horvath, Katja Adam, Perparim Kadriaj, Mihaela Kavran, Gregory L'Ambert, Riccardo P. Lia, Eduardo Marabuto, Raquel Medialdea Carrera, Rosario Melero-Alcibar, Antonios Michaelakis, Andrei Daniel Mihalca, Martina Micocci, Ognyan Mikov, Miguel A. Miranda, Pie Müller, Concepción Ornosa , Raimundo Outerelo, Domenico Otranto, Igor Pajovic, Javier Pérez-Tris, Dusan Petric, Maria Teresa Rebelo, Gilles Besnard, Elton Rogozi, AnaTello, Ángeles Vázquez , Marlen Vasquez, Toni Zitko, Francis Schaffner, Alessandra della Torre, and Joao Pinto

**Additional file 2: sequences obtained for the partial sequencing of domain III of the vgsc (voltage gated sodium channel gene) for a subsample of *Ae. albopictus* specimens included in the study.**

>BG20190016 Bulgaria Burgas

CAGGTGGGCAAGCAGCCGATTCGCGAGACCAACATCTACATGTACCTCTACTTYGTGTTCTTCATCATCTTCGGGTCGTTCTTCACCCTCAACCTGTTCATCGGTGTCATCATCGACAACTTCAACGAGCAGAAGAAGAAAGCCGGTGGCTCGCTGGAAATG

>20CH046 Switzerland Basel

CAGGTGGGCAAGCAGCCGATTCGCGAGACCAACATCTACATGTACCTCTACTTCGTGTTCTTCATCATCTTCGGGTCGTTCTTCACCCTCAACCTGTTCATCGGTGTCATCATCGACAACTTCAACGAGCAGAAGAAGAAAGCCGGTGGCTCGCTGGAAATG

>20CH048 Switzerland Basel

CAGGTGGGCAAGCAGCCRATTCGCGAGACCAACATCTACATGTACCTCTACTTCGTGTTCTTCATCATCTTCGGGTCGTTCTTCACCCTYAAYCTGTTCATCGGTGTCATCATCGACAACTTCAACGAGCAGAAGAAGAAAGCCGGTGGCTCGCTGGAAATG

>18_RO_349 Romania Gheorgheni

CAGGTGGGCAAGCAGCCGATTCGCGAGACCAACATCTACATGTACCTCTACTTCGTGTTCTTCATCATCTTCGGGTCGTTCTTCACCCTCAACCTGTTCATCGGTGTCATCATCGACAACTTCAACGAGCAGAAGAAGAAAGCCGGTGGCTCGCTGGAAATG

>19ME376 Montenegro Podgorica

CAGGTGGGCAAGCAGCCAATTCGCGAGACCAACATCTACATGTACCTCTACTTCGTGTTCTTCATCATCTTCGGGTCGTTCTTCACCCTCAACCTGTTCATCGGTGTCATCATCGACAACTTCAACGAGCAGAAGAAGAAAGCCGGTGGCTCGCTGGAAATG

>18_RS_241 Serbia Loznica

CAGGTGGGCAAGCAGCCGATTCGCGAGACCAACATCTACATGTACCTCTACTTYGTGTTCTTCATCATCTTCGGGTCGTTCTTCACCCTCAACCTGTTCATCGGTGTCATCATCGACAACTTCAACGAGCAGAAGAAGAAAGCCGGTGGCTCGCTGGAAATG

>BG20190017 Bulgaria Burgas

CAGGTGGGCAAGCAGCCRATTCGCGAGACCAACATCTACATGTACCTCTACTTCGTGTTCTTCATCATCTTCGGGTCGTTCTTCACCCTCAACCTGTTCATCGGTGTCATYATCGACAACTTCAACGAGCAGAAGAAGAAAGCCGGTGGCTCGCTGGAAATG

>20FR224 France Montpellier

CAGGTGGGCAAGCAGCCRATTCGCGAGACCAACATCTACATGTACCTCTACTTYGTGTTCTTCATCATCTTCGGGTCGTTCTTCACSCTCAACCTGTTCATCGGTGTCATCATCGACAACTTCAACGAGCAGAAGAAGAAAGCCGGTGGCTCGCTGGAAATG

>20FR017 France Nice

CAGGTGGGCAAGCAGCCGATTCGCGAGACCAACATCTACATGTACCTCTACTTCGTGTTCTTCATCATCTTCGGGTCGTTCTTCACCCTCAACCTGTTCATCGGTGTCATCATCGACAACTTCAACGAGCAGAAGAAGAAAGCCGGTGGCTCGCTGGAAATG

>19ME377 Montenegro Podgorica

CAGGTGGGCAAGCAGCCAATTCGCGAGACCAACATCTACATGTACCTCTACTTCGTGTTCTTCATCATCTTCGGGTCGTTCTTCACCCTCAACCTGTTCATCGGTGTCATCATCGACAACTTCAACGAGCAGAWGAAGAAAGCCGGTGGCTCGCTGGAAATG

>18_RO_353 Romania Gheorgheni

CAGGTGGGCAAGCAGCCRATTCGCGAGACCAACATCTACATGTACCTCTACTTCGTGTTCTTCATCATCTTCGGGTCGTTCTTCACCCTCAACCTGTTCATCGGTGTCATYATCGACAACTTCAMCGAGCAGAAGAAGAAAGCCGGTGGCTCGCTGGAAATG

>18_RS_244 Serbia Loznica

CAGGTGGGCAAGCAGCCAATTCGCGAGACCAACATCTACATGTACCTCTACTTCGTGTTCTTCATCATCTTCGGGTCGTTCTTCACCCYCAAYCTGTTCATCGGTGTCATCATCGACAACTTCAACGAGCAGAAGAAGAAAGCCGGTGGCTCGCTGGAAATG

>20CH067 Switzerland Coldrerio

CAGGTGGGCAAGCAGCCGATTCGCGAGACCAACATCTACATGTACCTCTACTTCGTGTTCTTCATCATCTTCGGGTCGTTCTTCACCCTCAACCTGTTCATCGGTGTCATCATCGACAACTTCAACGAGCAGAAGAAGAAAGCCGGTGGCTCGCTGGAAATG

>20FR003 France Nice

CAGGTGGGCAAGCAGCCAATTCGCGAGACCAACATCTACATGTACCTCTACTTCGTGTTCTTCATCATCTTCGGGTCGTTCTTCACCCTCAACCTGTTCATCGGTGTCATCATCGACAACTTCAACGAGCAGAAGAAGAAAGCCGGTGGCTCGCTGGAAATG

>20FR315 France Perpignan

CAGGTGGGCAAGCAGCCGATTCGCGAGACCAACATCTACATGTACCTCTACTTCGTGTTCTTCATCATCTTCGGGTCGTTCTTCACCCTCAACCTGTTCATCGGTGTCATCAKCGACAACTTCAACGAGCAGAAGAAGAAARMMGGTGGCTCGCTGGAAATG

>19ME378 Montenegro Podgorica

CAGGTGGGCAAGCAGCCGATTCGCGAGACCAACATCTACATGTACCTCTACTTCGTGTTCTTCATCATCTTCGGGTCGTTCTTCACCCTYAAYCTGTTCATCGGTGTCATCATCGACAACTTCAACGAGCAGAAGAAGAAAGCCGGTGGCTCGCTGGAAATG

>18_RO_356 Romania Gheorgheni

CAGGTGGGCAAGCAGCCRATTCGCGAGACCAACATCTACATGTACCTCTACTTCGTGTTCTTCATCATCTTCGGGTCGTTCTTCACCCTYAAYCTGTTCATCGGTGTCATCATCGACAACTTCAACGAGCAGAAGAAGAAAGCCGGTGGCTCGCTGGAAATG

>20CH001 Switzerland Basel

CAGGTGGGCAAGCAGCCGATTCGCGAGACCAACATCTACATGTACCTCTACTTCGTGTTCTTCATCATCTTCGGGTCGTTCTTCACCCTCAACCTGTTCATCGGTGTCATCATCGACAACTTCAACGAGCAGAAGAAGAAAGCCGGTGGCTCGCTGGAAATG

>BG20190003 Bulgaria Burgas

CAGGTGGGCAAGCAGCCGATTCGCGAGACCAACATCTACATGTACCTCTACTTCGTGTTCTTCATCATCTTCGGGTCGTTCTTCACCCTCAACCTGTTCATCGGTGTCATCATCGACAACTTCAACGAGCAGAAGAAGAAAGCCGGTGGCTCGCTGGAAATG

>20FR005 France Nice

CAGGTGGGCAAGCAGCCGATTCGCGAGACCAACATCTACATGTACCTCTACTTCGTGTTCTTCAYCATCTTCGGGTCGTTCTTCACCCTYAAYCTGTTCATCGGTGTCATCATCGACAACTTCAACGAGCAGAAGAAGAAAGCCGGTGGCTCGCTGGAAATG

>19ME361 Montenegro Podgorica

CAGGTGGGCAAGCAGCCGATTCGCGAGACCAACATCTACATGTACCTCTACTTCGTGTTCTTCATCATCTTCGGGTCGTTCTTCACCCTYAAYCTGTTCATCGGTGTCATCATCGACAACTTCAACGAGCAGAAGAAGAAAGCCGGTGGCTCGCTGGAAATG

>19ME385 Montenegro Podgorica

CAGGTGGGCAAGCAGCCRATTCGCGAGACCAACATCTACATGTACCTCTACTTCGTGTTCTTCATCATCTTCGGGTCGTTCTTCACCCTYAAYCTGTTCATCGGTGTCATCATCGACAACTTCAACGAGCAGAAGAAGAAAGCCGGTGGCTCGCTGGAAATG

>18_RS_233 Serbia Loznica

CAGGTGGGCAAGCAGCCGATTCGCGAGACCAACATCTACATGTACCTCTACTTYGTGTTCTTCATCATCTTCGGGTCGTTCTTCACCCTCAACCTGTTCATCGGTGTCATCATCGACAACTTCAACGAGCAGARGAAGAAAGCCGGTGGCTCGSTGGAAATG

>20CH003 Switzerland Basel

CAGGTGGGCAAGCAGCCGATTCGCGAGACCAACATCTACATGTACCTCTACTTYGTGTTCTTCATCATCTTCGGGTCGTTCTTCACSCTCAACCTGTTCATCGGTGTCATCATCGACAACTTCAACGAGCAGAAGAAGAAAGCCGGTGGCTCGCTGGAAATG

>BG20190009 Bulgaria Burgas

CAGGTGGGCAAGCAGCCGATTCGCGAGACCAACATCTACATGTACCTCTACTTCGTGTTCTTCATCATCTTCGGGTCGTTCTTCACCCTCAACCTGTTCATCGGTGTCATCATCGACAACTTCAACGAGCAGAAGAAGAAAGCCGGTGGCTCGCTGGAAATG

>20FR006 France Nice

CAGGTGGGCAAGCAGCCGATTCGCGAGACCAACATCTACATGTACCTCTACTTYGTGTTCTTCAYCATCTTCGGGTCGTTCTTCACCCTYAAYCTGTTCATCGGTGTCATCATCGACAACTTCAACGAGCAGAAGAAGAAAGCCGGTGGCTCGCTGGAAATG

>19ME362 Montenegro Podgorica

CAGGTGGGCAAGCAGCCRATTCGCGAGACCAACATCTACATGTACCTCTACTTCGTGTTCTTCATCATCTTCGGGTCGTTCTTCACCCTYAAYCTGTTCATCGGTGTCATCATCGACAACTTCAACGAGCAGAAGAAGAAAGCCGGTGGCTCGCTGGAAATG

>19ME386 Montenegro Podgorica

CAGGTGGGCAAGCAGCCAATTCGCGAGACCAACATCTACATGTACCTCTACTTCGTGTTCTTCATCATCTTCGGGTCGTTCTTCACCCTCAACCTGTTCATCGGTGTCATCATCGACAACTTCAACGAGCAGAAGAAGAAAGCCGGTGGCTCGCTGGAAATG

>18_RS_234 Serbia Loznica

CAGGTGGGCAAGCAGCCGATTCKCGAGACCAACATCTACATGTACCTCTACTTCGTGTTCTTCATCATCTTCGGGTCGTTCTTCACCCTCAACCTGTTCATCGGTGTCATCATCGACAACTTCAACGAGCAGA-----------------------------

>20CH038 Switzerland Basel

CAGGTGGGCAAGCAGCCGATTCGCGAGACCAACATCTACATGTACCTCTACTTYGTGTTCTTCATCATCTTCGGGTCGTTCTTCACCCTCAACCTGTTCATCGGTGTCATCATCGACAACTTCAACGAGCAGAAGAAGAAAGCCGGTGGCTCGCTGGAAATG

>20CH036 Switzerland Basel

CAGGTGGGCAAGCAGCCGATTCGCGAGACCAACATCTACATGTACCTCTACTTYGTGTTCTTCATCATCTTCGGGTCGTTCTTCACSCTCAACCTGTTCATCGGTGTCATCATCGACAACTTCAAMGAGCAGAAGAAGAAAGCCGGTGGCTCGCTGGAAATG

>20FR009 France Nice

CAGGTGGGCAAGCAGCCGATTCGCGAGACCAACATCTACATGTACCTCTACTTCGTGTTCTTCATCATCTTCGGGTCGTTCTTCACCCTCAACCTGTTCATCGGTGTCATCATCGACAACTTCAACGAGCAGAAGAAGAAAGCCGGTGGCTCGCTGGAAATG

>19ME365 Montenegro Podgorica

CAGGTGGGCAAGCAGCCGATTCGCGAGACCAACATCTACATGTACCTCTACTTCGTGTTCTTCATCATCTTCGGGTCGTTCTTCACCCTCAACCTGTTCATCGGTGTCATCATCGACAACTTCAACGAGCAGAAGAAGAAAGCCGGTGGCTCGCTGGAAATG

>18_RS_235 Serbia Loznica

CAGGTGGGCAAGCAGCCGATTCGCGAGACCAACATCTACATGTACCTCTACTTYGTGTTCTTCATCATCTTCGGGTCGTTCTTCACCCTCAACCTGTTCATCGGTGTCATCATCGACAACTTCAACGAGCAGAAGAAGAAAGCCGGTGGCTCGCTGGAAATG

>20CH039 Switzerland Basel

CAGGTGGGCAAGCAGCCGATTCGCGAGACCAACATCTACATGTACCTCTACTTCGTGTTCTTCATCATCTTCGGGTCGTTCTTCACCCTCAACCTGTTCATCGGTGTCATCATCGACAACTTCAACGAGCAGAAGAAGAAAGCCGGTGGCTCGCTGGAAATG

>20GR001 Italy Grosseto

CAGGTGGGCAAGCAGCCRATTCGCGAGACCAACATCTACATGTACCTCTACTTCGTGTTCTTCATCATCTTCGGGTCGTTCTTCACCCTCAACCTGTTCATCGGTGTCATYATCGACAACTTCAACGAGCAGAWGAAGAAAGCCGGTGGCTCGCTGGAAATG

>cpr alb 2 Cyprus Limassol

CAGGTGGGCAAGCAGCCGATCCGCGAGACCAACATCTACATGTACCTCTACTTTGTGTTCTTCATCATCTGCGGGTCGTTCTTCACCCTCAACCTGTTCATCGGTGTCATCATCGACAACTTCAACGAGCAGAAGAAGAAAGCCGGTGGCTCGCTGGAAATG

>cpr alb 4 Cyprus Limassol

CAGGTGGGCAAGCAGCCGATCCGCGAGACCAACATCTACATGTACCTCTACTTTGTGTTCTTCATCATCTGCGGGTCGTTCTTCACCCTCAACCTGTTCATCGGTGTCATCATCGACAACTTCAACGAGCAGAAGAAGAAAGCCGGTGGCTCGCTGGAAATG

>cpr alb 5 Cyprus Limassol

CAGGTGGGCAAGCAGCCGATCCGCGAGACCAACATCTACATGTACCTCTACTTTGTGTTCTTCATCATCTGCGGGTCGTTCTTCACCCTCAACCTGTTCATCGGTGTCATCATCGACAACTTCAACGAGCAGAAGAAGAAAGCCGGTGGCTCGCTGGAAATG

>cpr alb 6 Cyprus Limassol

CAGGTGGGCAAGCAGCCGATYCGCGAGACCAACATCTACATGTACCTCTACTTYGTGTTCTTCATCATCTKCGGGTCGTTCTTCACCCTCAACCTGTTCATCGGTGTCATCATCGACAACTTCAACGAGCAGAAGAAGAAAGCCGGTGGCTCGCTGGAAATG

>cpr alb 7 Cyprus Limassol

CAGGTGGGCAAGCAGCCGATYCGCGAGACCAACATCTACATGTACCTCTACTTYGTGTTCTTCATCATCTKCGGGTCGTTCTTCACCCTCAACCTGTTCATCGGTGTCATCATCGACAACTTCAACGAGCAGAAGAAGAAAGCCGGTGGCTCGCTGGAAATG

>cpr alb 8 Cyprus Limassol

CAGGTGGGCAAGCAGCCGATCCGCGAGACCAACATCTACATGTACCTCTACTTTGTGTTCTTCATCATCTGCGGGTCGTTCTTCACCCTCAACCTGTTCATCGGTGTCATCATCGACAACTTCAACGAGCAGAAGAAGAAAGCCGGTGGCTCGCTGGAAATG

>cpr alb 9 Cyprus Limassol

CAGGTGGGCAAGCAGCCGATYCGCGAGACCAACATCTACATGTACCTCTACTTYGTGTTCTTCATCATCTKCGGGTCGTTCTTCACCCTCAACCTGTTCATCGGTGTCATCATCGACAACTTCAACGAGCAGAAGAAGAAAGCCGGTGGCTCGCTGGAAATG

>cpr alb 10 Cyprus Limassol

CAGGTGGGCAAGCAGCCGATCCGCGAGACCAACATCTACATGTACCTCTACTTTGTGTTCTTCATCATCTGCGGGTCGTTCTTCACCCTCAACCTGTTCATCGGTGTCATCATCGACAACTTCAACGAGCAGAAGAAGAAAGCCGGTGGCTCGCTGGAAATG

>cpr alb 11 Cyprus Limassol

CAGGTGGGCAAGCAGCCGATCCGCGAGACCAACATCTACATGTACCTCTACTTTGTGTTCTTCATCATCTGCGGGTCGTTCTTCACCCTCAACCTGTTCATCGGTGTCATCATCGACAACTTCAACGAGCAGAAGAAGAAAGCCGGTGGCTCGCTGGAAATG

>cpr alb 12 Cyprus Limassol

CAGGTGGGCAAGCAGCCGATCCGCGAGACCAACATCTACATGTACCTCTACTTTGTGTTCTTCATCATCTGCGGGTCGTTCTTCACCCTCAACCTGTTCATCGGTGTCATCATCGACAACTTCAACGAGCAGAAGAAGAAAGCCGGTGGCTCGCTGGAAATG

>cpr alb 13 Cyprus Limassol

CAGGTGGGCAAGCAGCCGATYCGCGAGACCAACATCTACATGTACCTCTACTTYGTGTTCTTCATCATCTKCGGGTCGTTCTTCACCCTCAACCTGTTCATCGGTGTCATCATCGACAACTTCAACGAGCAGAAGAAGAAAGCCGGTGGCTCGCTGGAAATG

>cpr alb 14 Cyprus Limassol

CAGGTGGGCAAGCAGCCGATCCGCGAGACCAACATCTACATGTACCTCTACTTTGTGTTCTTCATCATCTGCGGGTCGTTCTTCACCCTCAACCTGTTCATCGGTGTCATCATCGACAACTTCAACGAGCAGAAGAAGAAAGCCGGTGGCTCGCTGGAAATG

>cpr alb 15 Cyprus Limassol

CAGGTGGGCAAGCAGCCGATCCGCGAGACCAACATCTACATGTACCTCTACTTTGTGTTCTTCATCATCTGCGGGTCGTTCTTCACCCTCAACCTGTTCATCGGTGTCATCATCGACAACTTCAACGAGCAGAAGAAGAAAGCCGGTGGCTCGCTGGAAATG

>cpr alb 16 Cyprus Limassol

CAGGTGGGCAAGCAGCCGATCCGCGAGACCAACATCTACATGTACCTCTACTTTGTGTTCTTCATCATCTGCGGGTCGTTCTTCACCCTCAACCTGTTCATCGGTGTCATCATCGACAACTTCAACGAGCAGAAGAAGAAAGCCGGTGGCTCGCTGGAAATG

>cpr alb 17 Cyprus Limassol

CAGGTGGGCAAGCAGCCGATCCGCGAGACCAACATCTACATGTACCTCTACTTTGTGTTCTTCATCATCTGCGGGTCGTTCTTCACCCTCAACCTGTTCATCGGTGTCATCATCGACAACTTCAACGAGCAGAAGAAGAAAGCCGGTGGCTCGCTGGAAATG

>cpr alb 19 Cyprus Limassol

CAGGTGGGCAAGCAGCCGATCCGCGAGACCAACATCTACATGTACCTCTACTTTGTGTTCTTCATCATCTGCGGGTCGTTCTTCACCCTCAACCTGTTCATCGGTGTCATCATCGACAACTTCAACGAGCAGAAGAAGAAAGCCGGTGGCTCGCTGGAAATG

>cpr alb 20 Cyprus Limassol

CAGGTGGGCAAGCAGCCGATCCGCGAGACCAACATCTACATGTACCTCTACTTTGTGTTCTTCATCATCTGCGGGTCGTTCTTCACCCTCAACCTGTTCATCGGTGTCATCATCGACAACTTCAACGAGCAGAAGAAGAAAGCCGGTGGCTCGCTGGAAATG

>cpr alb 21 Cyprus Limassol

CAGGTGGGCAAGCAGCCGATYCGCGAGACCAACATCTACATGTACCTCTACTTYGTGTTCTTCATCATCTKCGGGTCGTTCTTCACCCTCAACCTGTTCATCGGTGTCATCATCGACAACTTCAACGAGCAGAAGAAGAAAGCCGGTGGCTCGCTGGAAATG

>cpr alb 22 Cyprus Limassol

CAGGTGGGCAAGCAGCCGATCCGCGAGACCAACATCTACATGTACCTCTACTTTGTGTTCTTCATCATCTGCGGGTCGTTCTTCACCCTCAACCTGTTCATCGGTGTCATCATCGACAACTTCAACGAGCAGAAGAAGAAAGCCGGTGGCTCGCTGGAAATG

>cpr alb 24 Cyprus Limassol

CAGGTGGGCAAGCAGCCGATCCGCGAGACCAACATCTACATGTACCTCTACTTTGTGTTCTTCATCATCTGCGGGTCGTTCTTCACCCTCAACCTGTTCATCGGTGTCATCATCGACAACTTCAACGAGCAGAAGAAGAAAGCCGGTGGCTCGCTGGAAATG

>cpr alb 27 Cyprus Limassol

CAGGTGGGCAAGCAGCCGATCCGCGAGACCAACATCTACATGTACCTCTACTTTGTGTTCTTCATCATCTGCGGGTCGTTCTTCACCCTCAACCTGTTCATCGGTGTCATCATCGACAACTTCAACGAGCAGAAGAAGAAAGCCGGTGGCTCGCTGGAAATG

>cpr alb 28 Cyprus Limassol

CAGGTGGGCAAGCAGCCGATYCGCGAGACCAACATCTACATGTACCTCTACTTYGTGTTCTTCATCATCTKCGGGTCGTTCTTCACCCTCAACCTGTTCATCGGTGTCATCATCGACAACTTCAACGAGCAGAAGAAGAAAGCCGGTGGCTCGCTGGAAATG

>cpr alb 29 Cyprus Limassol

CAGGTGGGCAAGCAGCCGATCCGCGAGACCAACATCTACATGTACCTCTACTTTGTGTTCTTCATCATCTGCGGGTCGTTCTTCACCCTCAACCTGTTCATCGGTGTCATCATCGACAACTTCAACGAGCAGAAGAAGAAAGCCGGTGGCTCGCTGGAAATG

>cpr alb 30 Cyprus Limassol

CAGGTGGGCAAGCAGCCGATCCGCGAGACCAACATCTACATGTACCTCTACTTTGTGTTCTTCATCATCTGCGGGTCGTTCTTCACCCTCAACCTGTTCATCGGTGTCATCATCGACAACTTCAACGAGCAGAAGAAGAAAGCCGGTGGCTCGCTGGAAATG

>cpr alb 32 Cyprus Limassol

CAGGTGGGCAAGCAGCCGATYCGCGAGACCAACATCTACATGTACCTCTACTTYGTGTTCTTCATCATCTKCGGGTCGTTCTTCACCCTCAACCTGTTCATCGGTGTCATCATCGACAACTTCAACGAGCAGAAGAAGAAAGCCGGTGGCTCGCTGGAAATG

>cpr alb 33 Cyprus Limassol

CAGGTGGGCAAGCAGCCGATCCGCGAGACCAACATCTACATGTACCTCTACTTTGTGTTCTTCATCATCTGCGGGTCGTTCTTCACCCTCAACCTGTTCATCGGTGTCATCATCGACAACTTCAACGAGCAGAAGAAGAAAGCCGGTGGCTCGCTGGAAATG

>cpr alb 34 Cyprus Limassol

CAGGTGGGCAAGCAGCCGATCCGCGAGACCAACATCTACATGTACCTCTACTTTGTGTTCTTCATCATCTGCGGGTCGTTCTTCACCCTCAACCTGTTCATCGGTGTCATCATCGACAACTTCAACGAGCAGAAGAAGAAAGCCGGTGGCTCGCTGGAAATG

>cpr alb 35 Cyprus Limassol

CAGGTGGGCAAGCAGCCGATCCGCGAGACCAACATCTACATGTACCTCTACTTTGTGTTCTTCATCATCTGCGGGTCGTTCTTCACCCTCAACCTGTTCATCGGTGTCATCATCGACAACTTCAACGAGCAGAAGAAGAAAGCCGGTGGCTCGCTGGAAATG

>cpr alb 37 Cyprus Limassol

CAGGTGGGCAAGCAGCCGATCCGCGAGACCAACATCTACATGTACCTCTACTTTGTGTTCTTCATCATCTGCGGGTCGTTCTTCACCCTCAACCTGTTCATCGGTGTCATCATCGACAACTTCAACGAGCAGAAGAAGAAAGCCGGTGGCTCGCTGGAAATG

>cpr alb 38 Cyprus Limassol

CAGGTGGGCAAGCAGCCGATYCGCGAGACCAACATCTACATGTACCTCTACTTYGTGTTCTTCATCATCTKCGGGTCGTTCTTCACCCTCAACCTGTTCATCGGTGTCATCATCGACAACTTCAACGAGCAGAAGAAGAAAGCCGGTGGCTCGCTGGAAATG

>cpr alb 39 Cyprus Limassol

CAGGTGGGCAAGCAGCCGATCCGCGAGACCAACATCTACATGTACCTCTACTTTGTGTTCTTCATCATCTGCGGGTCGTTCTTCACCCTCAACCTGTTCATCGGTGTCATCATCGACAACTTCAACGAGCAGAAGAAGAAAGCCGGTGGCTCGCTGGAAATG

>cpr alb 40 Cyprus Limassol

CAGGTGGGCAAGCAGCCGATCCGCGAGACCAACATCTACATGTACCTCTACTTTGTGTTCTTCATCATCTGCGGGTCGTTCTTCACCCTCAACCTGTTCATCGGTGTCATCATCGACAACTTCAACGAGCAGAAGAAGAAAGCCGGTGGCTCGCTGGAAATG

>cpr alb 41 Cyprus Limassol

CAGGTGGGCAAGCAGCCGATCCGCGAGACCAACATCTACATGTACCTCTACTTTGTGTTCTTCATCATCTGCGGGTCGTTCTTCACCCTCAACCTGTTCATCGGTGTCATCATCGACAACTTCAACGAGCAGAAGAAGAAAGCCGGTGGCTCGCTGGAAATG

>cpr alb 43 Cyprus Limassol

CAGGTGGGCAAGCAGCCGATCCGCGAGACCAACATCTACATGTACCTCTACTTTGTGTTCTTCATCATCTGCGGGTCGTTCTTCACCCTCAACCTGTTCATCGGTGTCATCATCGACAACTTCAACGAGCAGAAGAAGAAAGCCGGTGGCTCGCTGGAAATG

>cpr alb 44 Cyprus Limassol

CAGGTGGGCAAGCAGCCGATCCGCGAGACCAACATCTACATGTACCTCTACTTTGTGTTCTTCATCATCTGCGGGTCGTTCTTCACCCTCAACCTGTTCATCGGTGTCATCATCGACAACTTCAACGAGCAGAAGAAGAAAGCCGGTGGCTCGCTGGAAATG

>cpr alb 45 Cyprus Limassol

CAGGTGGGCAAGCAGCCGATCCGCGAGACCAACATCTACATGTACCTCTACTTTGTGTTCTTCATCATCTGCGGGTCGTTCTTCACCCTCAACCTGTTCATCGGTGTCATCATCGACAACTTCAACGAGCAGAAGAAGAAAGCCGGTGGCTCGCTGGAAATG

>cpr alb 46 Cyprus Limassol

CAGGTGGGCAAGCAGCCGATYCGCGAGACCAACATCTACATGTACCTCTACTTYGTGTTCTTCATCATCTKCGGGTCGTTCTTCACCCTCAACCTGTTCATCGGTGTCATCATCGACAACTTCAACGAGCAGAAGAAGAAAGCCGGTGGCTCGCTGGAAATG

>cpr alb 47 Cyprus Limassol

CAGGTGGGCAAGCAGCCGATYCGCGAGACCAACATCTACATGTACCTCTACTTYGTGTTCTTCATCATCTKCGGGTCGTTCTTCACCCTCAACCTGTTCATCGGTGTCATCATCGACAACTTCAACGAGCAGAAGAAGAAAGCCGGTGGCTCGCTGGAAATG

>cpr alb 48 Cyprus Limassol

CAGGTGGGCAAGCAGCCGATCCGCGAGACCAACATCTACATGTACCTCTACTTTGTGTTCTTCATCATCTGCGGGTCGTTCTTCACCCTCAACCTGTTCATCGGTGTCATCATCGACAACTTCAACGAGCAGAAGAAGAAAGCCGGTGGCTCGCTGGAAATG

>cpr alb 50 Cyprus Limassol

CAGGTGGGCAAGCAGCCGATCCGCGAGACCAACATCTACATGTACCTCTACTTTGTGTTCTTCATCATCTGCGGGTCGTTCTTCACCCTCAACCTGTTCATCGGTGTCATCATCGACAACTTCAACGAGCAGAAGAAGAAAGCCGGTGGCTCGCTGGAAATG

>cpr alb 52 Cyprus Limassol

CAGGTGGGCAAGCAGCCGATYCGCGAGACCAACATCTACATGTACCTCTACTTYGTGTTCTTCATCATCTKCGGGTCGTTCTTCACCCTCAACCTGTTCATCGGTGTCATCATCGACAACTTCAACGAGCAGAAGAAGAAAGCCGGTGGCTCGCTGGAAATG

>cpr alb 53 Cyprus Limassol

CAGGTGGGCAAGCAGCCGATCCGCGAGACCAACATCTACATGTACCTCTACTTTGTGTTCTTCATCATCTGCGGGTCGTTCTTCACCCTCAACCTGTTCATCGGTGTCATCATCGACAACTTCAACGAGCAGAAGAAGAAAGCCGGTGGCTCGCTGGAAATG

>cpr alb 54 Cyprus Limassol

CAGGTGGGCAAGCAGCCGATYCGCGAGACCAACATCTACATGTACCTCTACTTYGTGTTCTTCATCATCTKCGGGTCGTTCTTCACCCTCAACCTGTTCATCGGTGTCATCATCGACAACTTCAACGAGCAGAAGAAGAAAGCCGGTGGCTCGCTGGAAATG

>ABAL02 Abkasia Gagra

CAGGTGGGCAAGCAGCCGATTCGCGAGACCAACATCTACATGTACCTCTACTTCGTGTTCTTCATCATCTTCGGGTCGTTCTTCACCCTCAACCTGTTCATCGGTGTCATCATCGACAACTTCAACGAGCAGAAGAAGAAAGCCGGTGGCTCGCTGGAAATG

>ABAL03 Abkasia Gagra

CAGGTGGGCAAGCAGCCGATTCGCGAGACCAACATCTACATGTACCTCTACTTCGTGTTCTTCATCATCTTCGGGTCGTTCTTCACCCTTAATCTGTTCATCGGTGTCATCATCGACAACTTCAACGAGCAGAAGAAGAAAGCCGGTGGCTCGCTGGAAATG

>ABGL02 Abkasia Gagra

CAGGTGGGCAAGCAGCCRATTCGCGAGACCAACATCTACATGTACCTCTACTTCGTGTTCTTCATCATCTTCGGGTCGTTCTTCACCCTCAACCTGTTCATCGGTGTCATCATCGACAACTTCAACGAGCAGAAGAAGAAAGCCGGTGGCTCGCTGGAAATG

>ABIN01 Abkasia Gagra

CAGGTGGGCAAGCAGCCRATTCGCGAGACCAACATCTACATGTACCTCTACTTCGTGTTCTTCATCATCTTCGGGTCGTTCTTCACCCTTAATCTGTTCATCGGTGTCATCATCGACAACTTCAACGAGCAGAAGAAGAAAGCCGGTGGCTCGCTGGAAATG

>ABIN02 Abkasia Gagra

CAGGTGGGCAAGCAGCCGATTCGCGAGACCAACATCTACATGTACCTCTACTTCGTGTTCTTCATCATCTTCGGGTCGTTCTTCACCCTCAACCTGTTCATCGGTGTCATCATCGACAACTTCAACGAGCAGAAGAAGAAAGCCGGTGGCTCGCTGGAAATG

>ABIN04 Abkasia Gagra

CAGGTGGGCAAGCAGCCGATTCGCGAGACCAACATCTACATGTACCTCTACTTCGTGTTCTTCATCATCTTCGGGTCGTTCTTCACCCTCAACCTGTTCATCGGTGTCATCATCGACAACTTCAACGAGCAGAAGAAGAAAGCCGGTGGCTCGCTGGAAATG

>ABIN05 Abkasia Gagra

CAGGTGGGCAAGCAGCCGATTCGCGAGACCAACATCTACATGTACCTCTACTTCGTGTTCTTCATCATCTTCGGGTCGTTCTTCACCCTCAACCTGTTCATCGGTGTCATCATCGACAACTTCAACGAGCAGAAGAAGAAAGCCGGTGGCTCGCTGGAAATG

>ALDU01 Albania Durres

CAGGTGGGCAAGCAGCCGATTCGCGAGACCAACATCTACATGTACCTCTACTTCGTGTTCTTCATCATCTTCGGGTCGTTCTTCACCCTTAATCTGTTCATCGGTGTCATCATCGACAACTTCAACGAGCAGAAGAAGAAAGCCGGTGGCTCGCTGGAAATG

>ALDU02 Albania Durres

CAGGTGGGCAAGCAGCCGATTCGCGAGACCAACATCTACATGTACCTCTACTTCGTGTTCTTCATCATCTTCGGGTCGTTCTTCACCCTCAACCTGTTCATCGGTGTCATCATCGACAACTTCAACGAGCAGAAGAAGAAAGCCGGTGGCTCGCTGGAAATG

>ALDU03 Albania Durres

CAGGTGGGCAAGCAGCCRATTCGCGAGACCAACATCTACATGTACCTCTACTTCGTGTTCTTCAYCATCTTCGGGTCGTTCTTCACCCTYAAYCTGTTCATCGGTGTCATCATCGACAACTTCAACGAGCAGAAGAAGAAAGCCGGTGGCTCGCTGGAAATG

>ALDU04 Albania Durres

CAGGTGGGCAAGCAGCCGATTCGCGAGACCAACATCTACATGTACCTCTACTTCGTGTTCTTCATCATCTTCGGGTCGTTCTTCACCCTCAACCTGTTCATCGGTGTCATCATCGACAACTTCAACGAGCAGAAGAAGAAAGCCGGTGGCTCGCTGGAAATG

>ALDU05 Albania Durres

CAGGTGGGCAAGCAGCCRATTCGCGAGACCAACATCTACATGTACCTCTACTTCGTGTTCTTCATCATCTTCGGGTCGTTCTTCACCCTCAACCTGTTCATCGGTGTCATCATCGACAACTTCAACGAGCAGAAGAAGAAAGCCGGTGGCTCGCTGGAAATG

>ALDU06 Albania Durres

CAGGTGGGCAAGCAGCCGATTCGCGAGACCAACATCTACATGTACCTCTACTTCGTGTTCTTCATCATCTTCGGGTCGTTCTTCACCCTCAACCTGTTCATCGGTGTCATCATCGACAACTTCAACGAGCAGAAGAAGAAAGCCGGTGGCTCGCTGGAAATG

>ALDU07 Albania Durres

CAGGTGGGCAAGCAGCCGATTCGCGAGACCAACATCTACATGTACCTCTACTTCGTGTTCTTCATCATCTTCGGGTCGTTCTTCACCCTCAACCTGTTCATCGGTGTCATCATCGACAACTTCAACGAGCAGAAGAAGAAAGCCGGTGGCTCGCTGGAAATG

>ALDU08 Albania Durres

CAGGTGGGCAAGCAGCCGATTCGCGAGACCAACATCTACATGTACCTCTACTTCGTGTTCTTCAYCATCTTCGGGTCGTTCTTCACCCTYAAYCTGTTCATCGGTGTCATCATCGACAACTTCAACGAGCAGAAGAAGAAAGCCGGTGGCTCGCTGGAAATG

>ALDU09 Albania Durres

CAGGTGGGCAAGCAGCCAATTCGCGAGACCAACATCTACATGTACCTCTACTTCGTGTTCTTCATCATCTTCGGGTCGTTCTTCACCCTYAAYCTGTTCATCGGTGTCATCATCGACAACTTCAACGAGCAGAAGAAGAAAGCCGGTGGCTCGCTGGAAATG

>ALDU10 Albania Durres

CAGGTGGGCAAGCAGCCGATTCGCGAGACCAACATCTACATGTACCTCTACTTCGTGTTCTTCATCATCTTCGGGTCGTTCTTCACCCTCAACCTGTTCATCGGTGTCATCATCGACAACTTCAACGAGCAGAAGAAGAAAGCCGGTGGCTCGCTGGAAATG

>ALDU11 Albania Durres

CAGGTGGGCAAGCAGCCGATTCGCGAGACCAACATCTACATGTACCTCTACTTCGTGTTCTTCATCATCTTCGGGTCGTTCTTCACCCTCAACCTGTTCATCGGTGTCATCATCGACAACTTCAACGAGCAGAAGAAGAAAGCCGGTGGCTCGCTGGAAATG

>ALDU12 Albania Durres

CAGGTGGGCAAGCAGCCGATTCGCGAGACCAACATCTACATGTACCTCTACTTCGTGTTCTTCATCATCTTCGGGTCGTTCTTCACCCTCAACCTGTTCATCGGTGTCATCATCGACAACTTCAACGAGCAGAAGAAGAAAGCCGGTGGCTCGCTGGAAATG

>ALDU13 Albania Durres

CAGGTGGGCAAGCAGCCGATTCGCGAGACCAACATCTACATGTACCTCTACTTYGTGTTCTTCATCATCTTCGGGTCGTTCTTCACCCTCAACCTGTTCATCGGTGTCATCATCGACAACTTCAACGAGCAGAAGAAGAAAGCCGGTGGCTCGCTGGAAATG

>ALDU14 Albania Durres

CAGGTGGGCAAGCAGCCGATTCGCGAGACCAACATCTACATGTACCTCTACTTCGTGTTCTTCATCATCTTCGGGTCGTTCTTCACCCTCAACCTGTTCATCGGTGTCATCATCGACAACTTCAACGAGCAGAAGAAGAAAGCCGGTGGCTCGCTGGAAATG

>ALDU15 Albania Durres

CAGGTGGGCAAGCAGCCGATTCGCGAGACCAACATCTACATGTACCTCTACTTCGTGTTCTTCATCATCTTCGGGTCGTTCTTCACCCTCAACCTGTTCATCGGTGTCATCATCGACAACTTCAACGAGCAGAAGAAGAAAGCCGGTGGCTCGCTGGAAATG

>ALDU16 Albania Durres

CAGGTGGGCAAGCAGCCRATTCGCGAGACCAACATCTACATGTACCTCTACTTCGTGTTCTTCATCATCTTCGGGTCGTTCTTCACCCTYAAYCTGTTCATCGGTGTCATCATCGACAACTTCAACGAGCAGAAGAAGAAAGCCGGTGGCTCGCTGGAAATG

>ALDU17 Albania Durres

CAGGTGGGCAAGCAGCCGATTCGCGAGACCAACATCTACATGTACCTCTACTTCGTGTTCTTCATCATCTTCGGGTCGTTCTTCACCCTCAACCTGTTCATCGGTGTCATCATCGACAACTTCAAMGAGCAGAAGAAGAAARCCGGTGGCTCGCTGGAAATG

>ALDU18 Albania Durres

CAGGTGGGCAAGCAGCCGATTCGCGAGACCAACATCTACATGTACCTCTACTTCGTGTTCTTCATCATCTTCGGGTCGTTCTTCACCCTCAACCTGTTCATCGGTGTCATCATCGACAACTTCAACGAGCAGAAGAAGAAAGCCGGTGGCTCGCTGGAAATG

>ALDU20 Albania Durres

CAGGTGGGCAAGCAGCCGATTCGCGAGACCAACATCTACATGTACCTCTACTTCGTGTTCTTCATCATCTTCGGGTCGTTCTTCACCCTCAACCTGTTCATCGGTGTCATCATCGACAACTTCAACGAGCAGAAGAAGAAAGCCGGTGGCTCGCTGGAAATG

>ALDU22 Albania Durres

CAGGTGGGCAAGCAGCCAATTCGCGAGACCAACATCTACATGTACCTCTACTTCGTGTTCTTCATCATCTTCGGGTCGTTCTTCACCCTCAAYCTGTTCATCGGTGTCATCATCGACAACTTCAACGAGCAGAAGAAGAAAGCCGGTGGCTCGCTGGAAATG

>ALKS01 Albania Saranda

CAGGTGGGCAAGCAGCCRATTCGCGAGACCAACATCTACATGTACCTCTACTTCGTGTTCTTCATCATCTTCGGGTCGTTCTTCACCCTYAAYCTGTTCATCGGTGTCATCATCGACAACTTCAACGAGCAGAAGAAGAAAGCCGGTGGCTCGCTGGAAATG

>ALKS03 Albania Saranda

CAGGTGGGCAAGCAGCCRATTCGCGAGACCAACATCTACATGTACCTCTACTTCGTGTTCTTCATCATCTTCGGGTCGTTCTTCACCCTYAAYCTGTTCATCGGTGTCATCATCGACAACTTCAACGAGCAGAAGAAGAAAGCCGGTGGCTCGCTGGAAATG

>ALKS04 Albania Saranda

CAGGTGGGCAAGCAGCCRATYCGCGAGACCAACATCTACATGTACCTCTACTTYGTGTTCTTCATCATCTKCGGGTCGTTCTTCACCCTYAAYCTGTTCATCGGTGTCATCATCGACAACTTCAACGAGCAGAAGAAGAAAGCCGGTGGCTCGCTGGAAATG

>ALKS05 Albania Saranda

CAGGTGGGCAAGCAGCCRATTCGCGAGACCAACATCTACATGTACCTCTACTTCGTGTTCTTCATCATCTTCGGGTCGTTCTTCACCCTYAAYCTGTTCATCGGTGTCATCATCGACAACTTCAACGAGCAGAAGAAGAAAGCCGGTGGCTCGCTGGAAATG

>ALKS06 Albania Saranda

CAGGTGGGCAAGCAGCCGATYCGCGAGACCAACATCTACATGTACCTCTACTTYGTGTTCTTCATCATCTKCGGGTCGTTCTTCACCCTYAAYCTGTTCATCGGTGTCATCATCGACAACTTCAACGAGCAGAAGAAGAAAGCCGGTGGCTCGCTGGAAATG

>ALKS14 Albania Saranda

CAGGTGGGCAAGCAGCCGATTCGCGAGACCAACATCTACATGTACCTCTACTTCGTGTTCTTCATCATCTTCGGGTCGTTCTTCACCCTCAACCTGTTCATCGGTGTCATCATCGACAACTTCAACGAGCAGAAGAAGAAAGCCGGTGGCTCGCTGGAAATG

>ALKS16 Albania Saranda

CAGGTGGGCAAGCAGCCGATTCGCGAGACCAACATCTACATGTACCTCTACTTCGTGTTCTTCATCATCTTCGGGTCGTTCTTCACCCTCAACCTGTTCATCGGTGTCATCATCGACAACTTCAACGAGCAGAAGAAGAAAGCCGGTGGCTCGCTGGAAATG

>ALKS34 Albania Saranda

CAGGTGGGCAAGCAGCCRATTCGCGAGACCAACATCTACATGTACCTCTACTTCGTGTTCTTCATCATCTTCGGGTCGTTCTTCACCCTYAAYCTGTTCATCGGTGTCATCATCGACAACTTCAACGAGCAGAAGAAGAAAGCCGGTGGCTCGCTGGAAATG

>BULO02 Bulgaria Lom

CAGGTGGGCAAGCAGCCGATTCGCGAGACCAACATCTACATGTACCTCTACTTCGTGTTCTTCATCATCTTCGGGTCGTTCTTCACCCTCAACCTGTTCATCGGTGTCATCATCGACAACTTCAACGAGCAGAAGAAGAAAGCCGGTGGCTCGCTGGAAATG

>ALKS17 Albania Saranda

CAGGTGGGCAAGCAGCCRATTCGCGAGACCAACATCTACATGTACCTCTACTTCGTGTTCTTCATCATCTTCGGGTCGTTCTTCACCCTYAAYCTGTTCATCGGTGTCATCATCGACAACTTCAACGAGCAGAAGAAGAAAGCCGGTGGCTCGCTGGAAATG

>BULO12 Bulgaria Lom

CAGGTGGGCAAGCAGCCRATTCGCGAGACCAACATCTACATGTACCTCTACTTCGTGTTCTTCATCATCTTCGGGTCGTTCTTCACCCTCAACCTGTTCATCGGTGTCATCATCGACAACTTCAACGAGCAGAAGAAGAAAGCCGGTGGCTCGCTGGAAATG

>BULO20 Bulgaria Lom

CAGGTGGGCAAGCAGCCGATTCGCGAGACCAACATCTACATGTACCTCTACTTYGTGTTCTTCATCATCTTCGGGTCGTTCTTCACCCTCAACCTGTTCATCGGTGTCATCATCGACAACTTCAACGAGCAGAAGAAGAAAGCCGGTGGCTCGCTGGAAATG

>BULO30 Bulgaria Lom

CAGGTGGGCAAGCAGCCGATTCGCGAGACCAACATCTACATGTACCTCTACTTCGTGTTCTTCATCATCTTCGGGTCGTTCTTCACCCTCAACCTGTTCATCGGTGTCATCATCGACAACTTCAACGAGCAGAAGAAGAAAGCCGGTGGCTCGCTGGAAATG

>BULO40 Bulgaria Lom

CAGGTGGGCAAGCAGCCGATTCGCGAGACCAACATCTACATGTACCTCTACTTCGTGTTCTTCATCATCTTCGGGTCGTTCTTCACCCTYAAYCTGTTCATCGGTGTCATCATCGACAACTTCAACGAGCAGAAGAAGAAAGCCGGTGGCTCGCTGGAAATG

>BULO42 Bulgaria Lom

CAGGTGGGCAAGCAGCCGATTCGCGAGACCAACATCTACATGTACCTCTACTTCGTGTTCTTCATCATCTTCGGGTCGTTCTTCACCCTYAAYCTGTTCATCGGTGTCATCATCGACAACTTCAACGAGCAGAAGAAGAAAGCCGGTGGCTCGCTGGAAATG

>CRPL02 Croatia Dubrovnik

CAGGTGGGCAAGCAGCCRATTCGCGAGACCAACATCTACATGTACCTCTACTTCGTGTTCTTCATCATCTTCGGGTCGTTCTTCACCCTYAAYCTGTTCATCGGTGTCATCATCGACAACTTCAACGAGCAGAAGAAGAAAGCCGGTGGCTCGCTGGAAATG

>CRPL03 Croatia Dubrovnik

CAGGTGGGCAAGCAGCCRATTCGCGAGACCAACATCTACATGTACCTCTACTTCGTGTTCTTCATCATCTTCGGGTCGTTCTTCACCCTCAACCTGTTCATCGGTGTCATCATCGACAACTTCAACGAGCAGAAGAAGAAAGCCGGTGGCTCGCTGGAAATG

>CRPL10 Croatia Dubrovnik

CAGGTGGGCAAGCAGCCRATTCGCGAGACCAACATCTACATGTACCTCTACTTCGTGTTCTTCATCATCTTCGGGTCGTTCTTCACCCTYAAYCTGTTCATCGGTGTCATCATCGACAACTTCAACGAGCAGAAGAAGAAAGCCGGTGGCTCGCTGGAAATG

>CRPL13 Croatia Dubrovnik

CAGGTGGGCAAGCAGCCRATTCGCGAGACCAACATCTACATGTACCTCTACTTCGTGTTCTTCATCATCTTCGGGTCGTTCTTCACCCTCAACCTGTTCATCGGTGTCATCATCGACAACTTCAACGAGCAGAAGAAGAAAGCCGGTGGCTCGCTGGAAATG

>CRPL15 Croatia Dubrovnik

CAGGTGGGCAAGCAGCCGATTCGCGAGACCAACATCTACATGTACCTCTACTTCGTGTTCTTCATCATCTTCGGGTCGTTCTTCACYCTCAACCTGTTCATCGGTGTCATCATCGACAACTTCAACGAGCAGAAGAAGAAAGCCGGTGGCTCGCTGGAAATG

>CRPL17 Croatia Dubrovnik

CAGGTGGGCAAGCAGCCGATTCGCGAGACCAACATCTACATGTACCTCTACTTCGTGTTCTTCATCATCTTCGGGTCGTTCTTCACCCTYAAYCTGTTCATCGGTGTCATCATCGACAACTTCAACGAGCAGAAGAAGAAAGCCGGTGGCTCGCTGGAAATG

>CRPL18 Croatia Dubrovnik

CAGGTGGGCAAGCAGCCGATTCGCGAGACCAACATCTACATGTACCTCTACTTCGTGTTCTTCATCATCTTCGGGTCGTTCTTCACCCTCAACCTGTTCATCGGTGTCATCATCGACAACTTCAACGAGCAGAAGAAGAAAGCCGGTGGCTCGCTGGAAATG

>CRPL19 Croatia Dubrovnik

CAGGTGGGCAAGCAGCCGATTCGCGAGACCAACATCTACATGTACCTCTACTTCGTGTTCTTCATCATCTTCGGGTCGTTCTTCACCCTCAACCTGTTCATCGGTGTCATCATCGACAACTTCAACGAGCAGAAGAAGAAAGCCGGTGGCTCGCTGGAAATG

>CRPL20 Croatia Dubrovnik

CAGGTGGGCAAGCAGCCGATTCGCGAGACCAACATCTACATGTACCTCTACTTCGTGTTCTTCATCATCTTCGGGTCGTTCTTCACCCTCAACCTGTTCATCGGTGTCATCATCGACAACTTCAACGAGCAGAAGAAGAAAGCCGGTGGCTCGCTGGAAATG

>CRPL40 Croatia Dubrovnik

CAGGTGGGCAAGCAGCCGATTCGCGAGACCAACATCTACATGTACCTCTACTTCGTGTTCTTCATCATCTTCGGGTCGTTCTTCACCCTCAACCTGTTCATCGGTGTCATCATCGACAACTTCAACGAGCAGAWGAAGAAAGCCGGTGGCTCGCTGGAAATG

>ESBA02 Spain Cornell del Llobregat

CAGGTGGGCAAGCAGCCRATTCGCGAGACCAACATCTACATGTACCTCTACTTCGTGTTCTTCATCATCTTCGGGTCGTTCTTCACCCTYAAYCTGTTCATCGGTGTCATCATCGACAACTTCAACGAGCAGAAGAAGAAAGCCGGTGGCTCGCTGGAAATG

>ESBA24 Spain Cornell del Llobregat

CAGGTGGGCAAGCAGCCGATTCGCGAGACCAACATCTACATGTACCTCTACTTCGTGTTCTTCATCATCTTCGGGTCGTTCTTCACCCTTAATCTGTTCATCGGTGTCATCATCGACAACTTCAACGAGCAGAAGAAGAAAGCCGGTGGCTCGCTGGAAATG

>ESBA25 Spain Cornell del Llobregat

CAGGTGGGCAAGCAGCCGATTCGCGAGACCAACATCTACATGTACCTCTACTTCGTGTTCTTCATCATCTTCGGGTCGTTCTTCACCCTCAACCTGTTCATCGGTGTCATCATCGACAACTTCAACGAGCAGAAGAAGAAAGCCGGTGGCTCGCTGGAAATG

>ESBS08 Spain Benicassim

CAGGTGGGCAAGCAGCCGATTCGCGAGACCAACATCTACATGTACCTCTACTTCGTGTTCTTCATCATCTTCGGGTCGTTCTTCACCCTCAACCTGTTCATCGGTGTCATCATCGACAACTTCAACGAGCAGAAGAAGAAAGCCGGTGGCTCGCTGGAAATG

>ESBY02 Spain Basauri

CAGGTGGGCAAGCAGCCAATTCGCGAGACCAACATCTACATGTACCTCTACTTCGTGTTCTTCATCATCTTCGGGTCGTTCTTCACCCTTAATCTGTTCATCGGTGTCATCATCGACAACTTCAACGAGCAGAAGAAGAAAGCCGGTGGCTCGCTGGAAATG

>ESBY04 Spain Basauri

CAGGTGGGCAAGCAGCCAATTCGCGAGACCAACATCTACATGTACCTCTACTTCGTGTTCTTCATCATCTTCGGGTCGTTCTTCACCCTTAATCTGTTCATCGGTGTCATCATCGACAACTTCAACGAGCAGAAGAAGAAAGCCGGTGGCTCGCTGGAAATG

>ESBY05 Spain Basauri

CAGGTGGGCAAGCAGCCAATTCGCGAGACCAACATCTACATGTACCTCTACTTCGTGTTCTTCATCATCTTCGGGTCGTTCTTCACCCTTAATCTGTTCATCGGTGTCATCATCGACAACTTCAACGAGCAGAAGAAGAAAGCCGGTGGCTCGCTGGAAATG

>ESBY06 Spain Basauri

CAGGTGGGCAAGCAGCCAATTCGCGAGACCAACATCTACATGTACCTCTACTTCGTGTTCTTCATCATCTTCGGGTCGTTCTTCACCCTTAATCTGTTCATCGGTGTCATCATCGACAACTTCAACGAGCAGAAGAAGAAAGCCGGTGGCTCGCTGGAAATG

>ESBY07 Spain Basauri

CAGGTGGGCAAGCAGCCAATTCGCGAGACCAACATCTACATGTACCTCTACTTCGTGTTCTTCATCATCTTCGGGTCGTTCTTCACCCTTAATCTGTTCATCGGTGTCATCATCGACAACTTCAACGAGCAGAAGAAGAAAGCCGGTGGCTCGCTGGAAATG

>ESBY08 Spain Basauri

CAGGTGGGCAAGCAGCCAATTCGCGAGACCAACATCTACATGTACCTCTACTTCGTGTTCTTCATCATCTTCGGGTCGTTCTTCACCCTTAATCTGTTCATCGGTGTCATCATCGACAACTTCAACGAGCAGAAGAAGAAAGCCGGTGGCTCGCTGGAAATG

>ESBY09 Spain Basauri

CAGGTGGGCAAGCAGCCAATTCGCGAGACCAACATCTACATGTACCTCTACTTCGTGTTCTTCATCATCTTCGGGTCGTTCTTCACCCTTAATCTGTTCATCGGTGTCATCATCGACAACTTCAACGAGCAGAAGAAGAAAGCCGGTGGCTCGCTGGAAATG

>ESBY13 Spain Basauri

CAGGTGGGCAAGCAGCCAATTCGCGAGACCAACATCTACATGTACCTCTACTTCGTGTTCTTCATCATCTTCGGGTCGTTCTTCACCCTTAATCTGTTCATCGGTGTCATCATCGACAACTTCAACGAGCAGAAGAAGAAAGCCGGTGGCTCGCTGGAAATG

>ESCP01 Spain Es Capdella

CAGGTGGGCAAGCAGCCRATTCGCGAGACCAACATCTACATGTACCTCTACTTCGTGTTCTTCATCATCTTCGGGTCGTTCTTYACYCTYAAYCTGTTCATCGGTGTCATCATCGACAACTTCAACGAGCAGAAGAAGAAAGCCGGTGGCTCGCTGGAAATG

>ESCP04 Spain Es Capdella

CAGGTGGGCAAGCAGCCGATTCGCGAGACCAACATCTACATGTACCTCTACTTCGTGTTCTTCATCATCTTCGGGTCGTTCTTCACCCTYAAYCTGTTCATCGGTGTCATCATCGACAACTTCAACGAGCAGAAGAAGAAAGCCGGTGGCTCGCTGGAAATG

>ESCP08 Spain Es Capdella

CAGGTGGGCAAGCAGCCGATTCGCGAGACCAACATCTACATGTACCTCTACTTCGTGTTCTTCATCATCTTCGGGTCGTTCTTCACCCTYAAYCTGTTCATCGGTGTCATCATCGACAACTTCAACGAGCAGAAGAAGAAAGCCGGTGGCTCGCTGGAAATG

>ESCP10 Spain Es Capdella

CAGGTGGGCAAGCAGCCGATTCGCGAGACCAACATCTACATGTACCTCTACTTCGTGTTCTTCATCATCTTCGGGTCGTTCTTCACCCTCAATCTGTTCATCGGTGTCATCATCGACAACTTCAACGAGCAGAAGAAGAAAGCCGGTGGCTCGCTGGAAATG

>ESCP15 Spain Es Capdella

CAGGTGGGCAAGCAGCCGATTCGCGAGACCAACATCTACATGTACCTCTACTTYGTGTTCTTCATCATCTTCGGGTCGTTCTTCACCCTYAAYCTGTTCATCGGTGTCATCATCGACAACTTCAACGAGCAGAAGAAGAAAGCCGGTGGCTCGCTGGAAATG

>ESCP16 Spain Es Capdella

CAGGTGGGCAAGCAGCCGATTCGCGAGACCAACATCTACATGTACCTCTACTTCGTGTTCTTCATCATCTTCGGGTCGTTCTTCACCCTYAAYCTGTTCATCGGTGTCATCATCGACAACTTCAACGAGCAGAAGAAGAAAGCCGGTGGCTCGCTGGAAATG

>ESCP17 Spain Es Capdella

CAGGTGGGCAAGCAGCCGATTCGCGAGACCAACATCTACATGTACCTCTACTTCGTGTTCTTCATCATCTTCGGGTCGTTCTTCACCCTYAAYCTGTTCATCGGTGTCATCATCGACAACTTCAACGAGCAGAAGAAGAAAGCCGGTGGCTCGCTGGAAATG

>ESCT04 Spain Cartagena

CAGGTGGGCAAGCAGCCGATTCGCGAGACCAACATCTACATGTACCTCTACTTCGTGTTCTTCACCATCTTCGGGTCGTTCTTCACCCTTAATCTGTTCATCGGTGTCATCATCGACAACTTCAACGAGCAGAAGAAGAAAGCCGGTGGCTCGCTGGAAATG

>ESLL06 Spain Cartagena

CAGGTGGGCAAGCAGCCAATTCGCGAGACCAACATCTACATGTACCTCTACTTCGTGTTCTTCATCATCTTCGGGTCGTTCTTCACCCTCAACCTGTTCATCGGTGTCATCATCGACAACTTCAACGAGCAGAAGAAGAAAGCCGGTGGCTCGCTGGAAATG

>ESLU02 Spain Cartagena

CAGGTGGGCAAGCAGCCGATTCGCGAGACCAACATCTACATGTACCTCTACTTCGTGTTCTTCATCATCTTCGGGTCGTTCTTCACCCTCAACCTGTTCATCGGTGTCATCATCGACAACTTCAACGAGCAGAAGAAGAAAGCCGGTGGCTCGCTGGAAATG

>ESMO04 Spain Monesterio

CAGGTGGGCAAGCAGCCGATTCGCGAGACCAACATCTACATGTACCTCTACTTCGTGTTCTTCATCATCTTCGGGTCGTTCTTCACCCTCAACCTGTTCATCGGTGTCATCATCGACAACTTCAACGAGCAGAAGAAGAAAGCCGGTGGCTCGCTGGAAATG

>ESSA01 Spain Cartagena

CAGGTGGGCAAGCAGCCAATTCGCGAGACCAACATCTACATGTACCTCTACTTCGTGTTCTTCATCATCTTCGGGTCGTTCTTCACCCTTAATCTGTTCATCGGTGTCATCATCGACAACTTCAACGAGCAGAAGAAGAAAGCCGGTGGCTCGCTGGAAATG

>ESTM02 Spain Torremolinos

CAGGTGGGCAAGCAGCCAATTCGCGAGACCAACATCTACATGTACCTCTACTTCGTGTTCTTCATCATCTTCGGGTCGTTCTTCACCCTTAATCTGTTCATCGGTGTCATCATCGACAACTTCAACGAGCAGAAGAAGAAAGCCGGTGGCTCGCTGGAAATG

>ESTO11 Spain Torremolinos

CAGGTGGGCAAGCAGCCGATTCGCGAGACCAACATCTACATGTACCTCTACTTCGTGTTCTTCATCATCTTCGGGTCGTTCTTCACCCTCAACCTGTTCATCGGTGTCATCATCGACAACTTCAACGAGCAGAAGAAGAAAGCCGGTGGCTCGCTGGAAATG

>ESTO14 Spain Torremolinos

CAGGTGGGCAAGCAGCCGATTCGCGAGACCAACATCTACATGTACCTCTACTTCGTGTTCTTCATCATCTTCGGGTCGTTCTTCACCCTCAACCTGTTCATCGGTGTCATCATCGACAACTTCAACGAGCAGAAGAAGAAAGCCGGTGGCTCGCTGGAAATG

>ESTY02 Spain Torremolinos

CAGGTGGGCAAGCAGCCGATTCGCGAGACCAACATCTACATGTACCTCTACTTCGTGTTCTTCATCATCTTCGGGTCGTTCTTCACCCTCAACCTGTTCATCGGTGTCATCATCGACAACTTCAACGAGCAGAAGAAGAAAGCCGGTGGCTCGCTGGAAATG

>ESTY05 Spain Torremolinos

CAGGTGGGCAAGCAGCCGATTCGCGAGACCAACATCTACATGTACCTCTACTTCGTGTTCTTCATCATCTTCGGGTCGTTCTTCACCCTCAACCTGTTCATCGGTGTCATCATCGACAACTTCAACGAGCAGAAGAAGAAAGCCGGTGGCTCGCTGGAAATG

>ESTY06 Spain Torremolinos

CAGGTGGGCAAGCAGCCGATTCGCGAGACCAACATCTACATGTACCTCTACTTCGTGTTCTTCATCATCTTCGGGTCGTTCTTCACCCTCAACCTGTTCATCGGTGTCATCATCGACAACTTCAACGAGCAGAAGAAGAAAGCCGGTGGCTCGCTGGAAATG

>ESTY08 Spain Torremolinos

CAGGTGGGCAAGCAGCCGATTCGCGAGACCAACATCTACATGTACCTCTACTTCGTGTTCTTCATCATCTTCGGGTCGTTCTTCACCCTCAACCTGTTCATCGGTGTCATCATCGACAACTTCAACGAGCAGAAGAAGAAAGCCGGTGGCTCGCTGGAAATG

>ESVL02 Spain Catarroja

CAGGTGGGCAAGCAGCCRATTCGCGAGACCAACATCTACATGTACCTCTACTTCGTGTTCTTCATCATCTTCGGGTCGTTCTTCACCCTYAAYCTGTTCATCGGTGTCATCATCGACAACTTCAACGAGCAGAAGAAGAAAGCCGGTGGCTCGCTGGAAATG

>ESVL04 Spain Catarroja

CAGGTGGGCAAGCAGCCRATTCGCGAGACCAACATCTACATGTACCTCTACTTYGTGTTCTTCATCATCTTCGGGTCGTTCTTCACCCTYAAYCTGTTCATCGGTGTCATCATCGACAACTTCAACGAGCAGAAGAAGAAAGCCGGTGGCTCGCTGGAAATG

>ESVL09 Spain Catarroja

CAGGTGGGCAAGCAGCCAATTCGCGAGACCAACATCTACATGTACCTCTACTTCGTGTTCTTCATCATCTTCGGGTCGTTCTTCACCCTTAATCTGTTCATCGGTGTCATCATCGACAACTTCAACGAGCAGAAGAAGAAAGCCGGTGGCTCGCTGGAAATG

>ESVL10 Spain Catarroja

CAGGTGGGCAAGCAGCCRATTCGCGAGACCAACATCTACATGTACCTCTACTTCGTGTTCTTCATCATCTTCGGGTCGTTCTTCACCCTYAAYCTGTTCATCGGTGTCATCATCGACAACTTCAACGAGCAGAAGAAGAAAGCCGGTGGCTCGCTGGAAATG

>ESVL11 Spain Catarroja

CAGGTGGGCAAGCAGCCAATTCGCGAGACCAACATCTACATGTACCTCTACTTCGTGTTCTTCATCATCTTCGGGTCGTTCTTCACCCTYAAYCTGTTCATCGGTGTCATCATCGACAACTTCAACGAGCAGAAGAAGAAAGCCGGTGGCTCGCTGGAAATG

>ESVL12 Spain Catarroja

CAGGTGGGCAAGCAGCCAATTCGCGAGACCAACATCTACATGTACCTCTACTTCGTGTTCTTCATCATCTTCGGGTCGTTCTTCACCCTTAATCTGTTCATCGGTGTCATCATCGACAACTTCAACGAGCAGAAGAAGAAAGCCGGTGGCTCGCTGGAAATG

>ESVL13 Spain Catarroja

CAGGTGGGCAAGCAGCCAATTCGCGAGACCAACATCTACATGTACCTCTACTTCGTGTTCTTCATCATCTTCGGGTCGTTCTTCACCCTYAAYCTGTTCATCGGTGTCATCATCGACAACTTCAACGAGCAGAAGAAGAAAGCCGGTGGCTCGCTGGAAATG

>ESVL16 Spain Catarroja

CAGGTGGGCAAGCAGCCAATTCGCGAGACCAACATCTACATGTACCTCTACTTCGTGTTCTTCATCATCTTCGGGTCGTTCTTCACCCTTAATCTGTTCATCGGTGTCATCATCGACAACTTCAACGAGCAGAAGAAGAAAGCCGGTGGCTCGCTGGAAATG

>ESVL20 Spain Catarroja

CAGGTGGGCAAGCAGCCGATTCGCGAGACCAACATCTACATGTACCTCTACTTCGTGTTCTTCATCATCTTCGGGTCGTTCTTCACCCTCAACCTGTTCATCGGTGTCATCATCGACAACTTCAACGAGCAGAAGAAGAAAGCCGGTGGCTCGCTGGAAATG

>FRMH05 France Saint-Martin-d'H res

--------------------------------------------CCTCTACTTCGTGTTCTTCATCATCTTCGGGTCGTTCTTCACCCTCAACCTGTTCATCGGTGTCATCATCGACAACTTCAACGAGCAGAAGAAGAAAGCCGGTGGCTCGCTGGAAATG

>FRMH06 France Saint-Martin-d'H res

-AGGTGGGCAAGCAGCCRATTCGCGAGACCAACATCTACATGTACCTCTACTTYGTGTTCTTCATCATCTTCGGGTCGTTCTTCACCCTYAAYCTGTTCATCGGTGTCATCATCGACAACTTCAACGAGCAGAAGAAGAAAGCCGGTGGCTCGCTGGAAATG

>FRMH20 France Saint-Martin-d'H res

CAGGTGGGCAAGCAGCCRATTCGCGAGACCAACATCTACATGTACCTCTACTTCGTGTTCTTCATCATCTTCGGGTCGTTCTTCACCCTYAAYCTGTTCATCGGTGTCATCATCGACAACTTCAACGAGCAGAAGAAGAAAGCCGGTGGCTCGCTGGAAATG

>FRST1.02 France ST-Bischeim

CAGGTGGGCAAGCAGCCRATTCGCGAGACCAACATCTACATGTACCTCTACTTCGTGTTCTTCATCATCTTCGGGTCGTTCTTCACCCTCAACCTGTTCATCGGTGTCATCATCGACAACTTCAACGAGCAGAAGAAGAAAGCCGGTGGCTCGCTGGAAATG

>FRST09.03 France ST-Hoenheim

CAGGTGGGCAAGCAGCCGATTCGCGAGACCAACATCTACATGTACCTCTACTTCGTGTTCTTCATCATCTTCGGGTCGTTCTTCACTCTCAACCTGTTCATCGGTGTCATCATCGACAACTTCAACGAGCAGAAGAAGAAAGCCGGTGGCTCGCTGGAAATG

>FRST10.02 France ST-Hoenheim

CAGGTGGGCAAGCAGCCGATTCGCGAGACCAACATCTACATGTACCTCTACTTCGTGTTCTTCATCATCTTCGGGTCGTTCTTCACYCTCAACCTGTTCATCGGTGTCATCATCGACAACTTCAACGAGCAGAAGAAGAAAGCCGGTGGCTCGCTGGAAATG

>FRST35.02 France ST-Strasbourg

CAGGTGGGCAAGCAGCCAATTCGCGAGACCAACATCTACATGTACCTCTACTTCGTGTTCTTCATCATCTTCGGGTCGTTCTTCACCCTTAATCTGTTCATCGGTGTCATCATCGACAACTTCAACGAGCAGAAGAAGAAAGCCGGTGGCTCGCTGGAAATG

>FRST35.04 France ST-Strasbourg

CAGGTGGGCAAGCAGCCRATTCGCGAGACCAACATCTACATGTACCTCTACTTCGTGTTCTTCAYCATCTTCGGGTCGTTCTTCACCCTTAATCTGTTCATCGGTGTCATCATCGACAACTTCAACGAGCAGAAGAAGAAAGCCGGTGGCTCGCTGGAAATG

>FRST35.05 France ST-Strasbourg

CAGGTGGGCAAGCAGCCRATTCGCGAGACCAACATCTACATGTACCTCTACTTCGTGTTCTTCATCATCTTCGGGTCGTTCTTCACCCTYAAYCTGTTCATCGGTGTCATCATCGACAACTTCAACGAGCAGAAGAAGAAAGCCGGTGGCTCGCTGGAAATG

>GRAA02 Greece Athens(AIA)

CAGGTGGGCAAGCAGCCGATYCGCGAGACCAACATCTACATGTACCTCTACTTYGTGTTCTTCATCATCTKCGGGTCGTTCTTCACCCTCAACCTGTTCATCGGTGTCATCATCGACAACTTCAACGAGCAGAAGAAGAAAGCCGGTGGCTCGCTGGAAAT-

>GRAA05 Greece Athens(AIA)

CAGGTGGGCAAGCAGCCGATTCGCGAGACCAACATCTACATGTACCTCTACTTYGTGTTCTTCATCATCTTCGGGTCGTTCTTCACCCTCAACCTGTTCATCGGTGTCATCATCGACAACTTCAACGAGCAGAAGAAGAAAGCCGGTGGCTCGCTGGAAATG

>GRAA07 Greece Athens(AIA)

CAGGTGGGCAAGCAGCCGATTCGCGAGACCAACATCTACATGTACCTCTACTTYGTGTTCTTCATCATCTTCGGGTCGTTCTTCACCCTCAACCTGTTCATCGGTGTCATCATCGACAACTTCAACGAGCAGAAGAAGAAAGCCGGTGGCTCGCTGGAAATG

>GRAA08 Greece Athens(AIA)

CAGGTGGGCAAGCAGCCGATCCGCGAGACCAACATCTACATGTACCTCTACTTYGTGTTCTTCATCATCTKCGGGTCGTTCTTCACCCTCAACCTGTTCATCGGTGTCATCATCGACAACTTCAACGAGCAGAAGAAGAAAGCCGGTGGCTCGCTGGAAATG

>GRAA09 Greece Athens(AIA)

CAGGTGGGCAAGCAGCCGATYCGCGAGACCAACATCTACATGTACCTCTACTTYGTGTTCTTCATCATCTKCGGGTCGTTCTTCACCCTCAACCTGTTCATCGGTGTCATCATCGACAACTTCAACGAGCAGAAGAAGAAAGCCGGTGGCTCGCTGGAAATG

>GRAA11 Greece Athens(AIA)

CAGGTGGGCAAGCAGCCGATTCGCGAGACCAACATCTACATGTACCTCTACTTYGTGTTCTTCATCATCTTCGGGTCGTTCTTCACCCTCAACCTGTTCATCGGTGTCATCATCGACAACTTCAACGAGCAGAAGAAGAAAGCCGGTGGCTCGCTGGAAATG

>GRAE01 Greece Athens(AIA)

CAGGTGGGCAAGCAGCCGATYCGCGAGACCAACATCTACATGTACCTCTACTTTGTGTTCTTCATCATCTKCGGGTCGTTCTTCACCCTCAACCTGTTCATYGGTGTCATCATCGACAACTTCAACGAGCAGAAGAAGAAAGCCGGTGGCTCKCTGGAAATG

>GRAE05 Greece Athens(AIA)

CAGGTGGGCAAGCAGCCGATYCGCGAGACCAACATCTACATGTACCTCTACTTYGTGTTCTTCAYCATCTKCGGGTCGTTCTTCACCCTYAAYCTGTTCATCGGTGTCATCATCGACAACTTCAACGAGCAGAAGAAGAAAGCCGGTGGCTCGCTGGAAATG

>GRAE07 Greece Athens(AIA)

CAGGTGGGCAAGCAGCCGATYCGCGAGACCAACATCTACATGTACCTCTACTTTGTGTTCTTCATCATCTKCGGGTCGTTCTTCACCCTCAACCTGTTCATCGGTGTCATCATCGACAACTTCAACGAGCAGAAGAAGAAAGCCGGTGGCTCGCTGGAAATG

>GRAE13 Greece Athens(AIA)

CAGGTGGGCAAGCAGCCGATYCGCGAGACCAACATCTACATGTACCTCTACTTYGTGTTCTTCATCATCTKCGGGTCGTTCTTCACCCTYAAYCTGTTCATCGGTGTCATCATCGACAACTTCAACGAGCAGAAGAAGAAAGCCGGTGGCTCGCTGGAAATG

>GRAE14 Greece Athens(AIA)

CAGGTKGGCAAGCAGCCRATTCGCGAGACCAACATCTACATGTACCTCTACTTYGTGTTCTTCATCATCTTCGGGTCGTTCTTCACCCTCAACCTGTTCATCGGTGTCATCATCGACAACTTCAACGAGCAGAAGAAGAAAGCCGGTGGCTCGCTGGAAATG

>GRAH01 Greece Athens(AIA)

CAGGTGGGCAAGCAGCCGATYCGCGAGACCAACATCTACATGTACCTCTACTTYGTGTTCTTCAYCATCTKCGGGTCGTTCTTCACCCTYAAYCTGTTCATCGGTGTCATCATCGACAACTTCAACGAGCAGAAGAAGAAAGCCGGTGGCTCGCTGGAAATG

>GRAH02 Greece Athens(AIA)

CAGGTGGGCAAGCAGCCGATYCGCGAGACCAACATCTACATGTACCTCTACTTTGTGTTCTTCATCATCTKCGGGTCGTTCTTCACCCTCAACCTGTTCATYGGTGTCATCATCGACAACTTCAACGAGCAGAAGAAGAAAGCCGGTGGCTCKCTGGAAATG

>GRAH05 Greece Athens(AIA)

CAGGTGGGCAAGCAGCCGATYCGCGAGACCAACATCTACATGTACCTCTACTTYGTGTTCTTCATCATCTKCGGGTCGTTCTTCACCCTCAACCTGTTCATCGGTGTCATCATCGACAACTTCAACGAGCAGAAGAAGAAAGCCGGTGGCTCGCTGGAAATG

>GRAH06 Greece Athens(AIA)

CAGGTGGGCAAGCAGCCGATYCGCGAGACCAACATCTACATGTACCTCTACTTYGTGTTCTTCATCATCTKCGGGTCGTTCTTCACCCTCAACCTGTTCATCGGTGTCATCATCGACAACTTCAACGAGCAGAAGAAGAAAGCCGGTGGCTCGCTGGAAATG

>GRAH07 Greece Athens(AIA)

CAGGTGGGCAAGCAGCCGATYCGCGAGACCAACATYTACATGTACCTYTACTTTGTGTTCTTCATCATYTKCGGGTCGTTCTTCACCCTCAACCTGTTCATCGGTGTCATCATCGACAACTTCAACGAGCAGAAGAAGAAAGCCGGTGGCTCGCTGGAAATG

>GRAI01 Greece Athens(AIA)

CAGGTGGGCAAGCAGCCGATYCGCGAGACCAACATCTACATGTACCTCTACTTYGTGTTCTTCATCATCTKCGGGTCGTTCTTCACCCTCAACCTGTTCATCGGTGTCATCATCGACAACTTCAACGAGCAGAAGAAGAAAGCCGGTGGCTCGCTGGAAATG

>GRAN03 Greece Athens(AIA)

CAGGTKGGCAAGCAGCCGATTCGCGAGACCAACATCTACATGTACCTCTACTTYGTGTTCTTCAYCATCTTCGGGTCGTTCTTCACCCTYAAYCTGTTCATCGGTGTCATCATCGACAACTTCAACGAGCAGAAGAAGAAAGCCGGTGGCTCGCTGGAAATG

>GRAN04 Greece Athens(AIA)

CAGGTGGGCAAGCAGCCGATYCGCGAGACCAACATCTACATGTACCTCTACTTTGTGTTCTTCATCATCTKCGGGTCGTTCTTCACCCTCAACCTGTTCATCGGTGTCATCATCGACAACTTCAACGAGCAGAAGAAGAAAGCCGGTGGCTCKCTGGAAATG

>GRAN06 Greece Athens(AIA)

CAGGTGGGCAAGCAGCCGATYCGCGAGACCAACATCTACATGTACCTCTACTTTGTGTTCTTCATCATCTKCGGGTCGTTCTTCACCCTCAACCTGTTCATCGGTGTCATCATCGACAACTTCAACGAGCAGAAGAAGAAAGCCGGTGGCTCKCTGGAAATG

>GRAN07 Greece Athens(AIA)

CAGGTGGGCAAGCAGCCGATTCGCGAGACCAACATCTACATGTACCTCTACTTCGTGTTCTTCATCATCTTCGGGTCGTTCTTCACCCTTAATCTGTTCATCGGTGTCATCATCGACAACTTCAACGAGCAGAAGAAGAAAGCCGGTGGCTCGCTGGAAATG

>GRAN08 Greece Athens(AIA)

CAGGTGGGCAAGCAGCCGATTCGCGAGACCAACATCTACATGTACCTCTACTTCGTGTTCTTCACCATCTTCGGGTCGTTCTTCACCCTTAATCTGTTCATCGGTGTCATCATCGACAACTTCAACGAGCAGAAGAAGAAAGCCGGTGGCTCGCTGGAAATG

>GRAN10 Greece Athens(AIA)

CAGGTGGGCAAGCAGCCGATYCGCGAGACCAACATCTACATGTACCTCTACTTTGTGTTCTTCAYCATCTKCGGGTCGTTCTTCACCCTCAACCTGTTCATCGGTGTCATCATCGACAACTTCAACGAGCAGAAGAAGAAAGCCGGTGGCTCGCTGGAAATG

>GRAN13 Greece Athens(AIA)

CAGGTGGGCAAGCAGCCGATYCGCGAGACCAACATCTACATGTACCTCTACTTTGTGTTCTTCATCATCTKCGGGTCGTTCTTCACCCTCAACCTGTTCATCGGTGTCATCATCGACAACTTCAACGAGCAGAAGAAGAAAGCCGGTGGCTCGCTGGAAATG

>GRAN15 Greece Athens(AIA)

CAGGTGGGCAAGCAGCCGATYCGCGAGACCAACATCTACATGTACCTCTACTTTGTGTTCTTCATCATCTKCGGGTCGTTCTTCACCCTCAACCTGTTCATCGGTGTCATCATCGACAACTTCAACGAGCAGAAGAAGAAAGCCGGTGGCTCGCTGGAAATG

>GRAN17 Greece Athens(AIA)

CAGGTGGGCAAGCAGCCGATYCGCGAGACCAACATCTACATGTACCTCTACTTYGTGTTCTTCATCATCTKCGGGTCGTTCTTCACCCTCAACCTGTTCATCGGTGTCATCATCGACAACTTCAACGAGCAGAAGAAGAAAGCCGGTGGCTCGCTGGAAATG

>GRCA01 Greece Chania

CAGGTGGGCAAGCAGCCGATTCGCGAGACCAACATCTACATGTACCTCTACTTCGTGTTCTTCATCATCTTCGGGTCGTTCTTCACCCTYAAYCTGTTCATCGGTGTCATCATCGACAACTTCAACGAGCAGAAGAAGAAAGCCGGTGGCTCGCTGGAAATG

>GRCA03 Greece Chania

CAGGTGGGCAAGCAGCCRATTCGCGAGACCAACATCTACATGTACCTCTACTTYGTGTTCTTCATCATCTTCGGGTCGTTCTTCACCCTYAAYCTGTTCATYGGTGTCATCATCGACAACTTCAACGAGCAGAAGAAGAAAGCCGGTGGCTCKCTGGAAATG

>GRCA04 Greece Chania

CAGGTGGGCAAGCAGCCGATTCGCGAGACCAACATCTACATGTACCTCTACTTYGTGTTCTTCATCATCTTCGGGTCGTTCTTCACCCTCAACCTGTTCATCGGTGTCATCATCGACAACTTCAACGAGCAGAAGAAGAAAGCCGGTGGCTCGCTGGAAATG

>GRCA05 Greece Chania

CAGGTGGGCAAGCAGCCRATTCGCGAGACCAACATCTACATGTACCTCTACTTCGTGTTCTTCATCATCTTCGGGTCGTTCTTCACCCTYAAYCTGTTCATCGGTGTCATCATCGACAACTTCAACGAGCAGAAGAAGAAAGCCGGTGGCTCGCTGGAAATG

>GRCC01 Greece Chania

CAGGTGGGCAAGCAGCCGATYCGCGAGACCAACATCTACATGTACCTCTACTTYGTGTTCTTCATCATCTKCGGGTCGTTCTTCACCCTCAACCTGTTCATCGGTGTCATCATCGACAACTTCAACGAGCAGAAGAAGAAAGCCGGTGGCTCGCTGGAAATG

>GRCC03 Greece Chania

CAGGTGGGCAAGCAGCCGATYCGCGAGACCAACATCTACATGTACCTCTACTTYGTGTTCTTCATCATCTKCGGGTCGTTCTTCACCCTCAACCTGTTCATCGGTGTCATCATCGACAACTTCAACGAGCAGAAGAAGAAAGCCGGTGGCTCGCTGGAAATG

>GRCC04 Greece Chania

CAGGTGGGCAAGCAGCCRATTCGCGAGACCAACATCTACATGTACCTCTACTTCGTGTTCTTCATCATCTTCGGGTCGTTCTTCACCCTYAAYCTGTTCATCGGTGTCATCATCGACAACTTCAACGAGCAGAAGAAGAAAGCCGGTGGCTCGCTGGAAATG

>GRCC05 Greece Chania

CAGGTGGGCAAGCAGCCRATTCGCGAGACCAACATCTACATGTACCTCTACTTYGTGTTCTTCATCATCTTCGGGTCGTTCTTCACCCTYAAYCTGTTCATCGGTGTCATCATCGACAACTTCAACGAGCAGAAGAAGAAAGCCGGTGGCTCGCTGGAAATG

>GRCC06 Greece Chania

CAGGTGGGCAAGCAGCCRATTCGCGAGACCAACATCTACATGTACCTCTACTTCGTGTTCTTCATCATCTTCGGGTCGTTCTTCACCCTCAACCTGTTYATCGGTGTCATCATCGACAACTTCAACGAGCAGAAGAAGAAAGCCGGTGGCTCGC-GGAAATG

>GRCC07 Greece Chania

CAGGTGGGCAAGCAGCCGATTCGCGAGACCAACATCTACATGTACCTCTACTTTGTGTTCTTCATCATCTTCGGGTCGTTCTTCACCCTCAATCTGTTCATCGGTGTCATCATCGACAACTTCAACGAGCAGAAGAAGAAGGCCGGCGGCTCGCTAGAA---

>GRCC08 Greece Chania

CAGGTGGGCAAGCAGCCGATTCGCGAGACCAACATCTACATGTACCTCTACTTCGTGTTCTTCATCATCTTCGGGTCGTTCTTCACCCTCAACCTGTTCATCGGTGTCATCATCGACAACTTCAACGAGCAGAAGAAGAAAGCCGGTGGCTCGCTGGAAATG

>GRCC09 Greece Chania

CAGGTGGGCAAGCAGCCGATTCGCGAGACCAACATCTACATGTACCTCTACTTYGTGTTCTTCATCATCTTCGGGTCGTTCTTCACCCTCAACCTGTTCATYGGTGTCATCATCGACAACTTCAACGAGCAGAAGAAGAAAGCCGGTGGCTCKCTGGAAATG

>GRCC10 Greece Chania

CAGGTGGGCAAGCAGCCGATTCGCGAGACCAACATCTACATGTACCTCTACTTYGTGTTCTTCATCATCTTCGGGTCGTTCTTCACCCTCAACCTGTTCATCGGTGTCATCATCGACAACTTCAACGAGCAGAAGAAGAAAGCCGGTGGCTCGCTGGAAATG

>GRCC13 Greece Chania

CAGGTGGGCAAGCAGCCRATTCGCGAGACCAACATCTACATGTACCTCTACTTCGTGTTCTTCATCATCTTCGGGTCGTTCTTCACCCTYAAYCTGTTCATCGGTGTCATCATCGACAACTTCAACGAGCAGAAGAAGAAAGCCGGTGGCTCGCTGGAAATG

>GRCC15 Greece Chania

CAGGTGGGCAAGCAGCCGATTCGCGAGACCAACATCTACATGTACCTCTACTTCGTGTTCTTCATCATCTTCGGGTCGTTCTTCACCCTCAACCTGTTCATCGGTGTCATCATCGACAACTTCAACGAGCAGAAGAAGAAAGCCGGTGGCTCGCTGGAAATG

>GRCC16 Greece Chania

CAGGTGGGCAAGCAGCCGATTCGCGAGACCAACATCTACATGTACCTCTACTTCGTGTTCTTCATCATCTTCGGGTCGTTCTTCACCCTCAACCTGTTCATCGGTGTCATCATCGACAACTTCAACGAGCAGAAGAAGAAAGCCGGTGGCTCGCTGGAAATG

>GRCC17 Greece Chania

CAGGTGGGCAAGCAGCCGATYCGCGAGACCAACATCTACATGTACCTCTACTTYGTGTTCTTCATCATCTKCGGGTCGTTCTTCACCCTCAACCTGTTCATCGGTGTCATCATCGACAACTTCAACGAGCAGAAGAAGAAAGCCGGTGGCTCGCTGGAAATG

>GRCC20 Greece Chania

CAGGTGGGCAAGCAGCCGATTCGCGAGACCAACATCTACATGTACCTCTACTTCGTGTTCTTCATCATCTTCGGGTCGTTCTTCACCCTCAACCTGTTCATCGGTGTCATCATCGACAACTTCAACGAGCAGAAGAAGAAAGCCGGTGGCTCGCTGGAAATG

>GRCC21 Greece Chania

CAGGTGGGCAAGCASCCRATWCGCGAGWCCAACATCTACATGTACCTCTACTTCGTGTTCTTCATCATCTTCGGGTCGTTCTTCACCCTCAACCTGTTCATCGGTGTCATCATCGACAACTTCAACGAGCAGAAGAAGAAAGCCGGTGGCTCGCTGGAAATG

>GRCC22 Greece Chania

CAGGTGGGCAAGCAGCCGATYCGCGAGACCAACATCTACATGTACCTCTACTTYGTGTTCTTCATCATCTKCGGGTCGTTCTTCACCCTCAACCTGTTCATCGGTGTCATCATCGACAACTTCAACGAGCAGAAGAAGAAAGCCGGTGGCTCGCTGGAAATG

>GRCC24 Greece Chania

CAGGTGGGCAAGCAGCCRATYCGCGAGACCAACATCTACATGTACCTCTACTTYGTGTTCTTCATCATCTKCGGGTCGTTCTTCACCCTYAAYCTGTTCATCGGTGTCATCATCGACAACTTCAACGAGCAGAAGAAGAAAGCCGGTGGCTCGCTGGAAATG

>GRCH01 Greece Chania

CAGGTGGGCAAGCAGCCGATTCGCGAGACCAACATCTACATGTACCTCTACTTTGTGTTCTTCATCATCTTCGGGTCGTTCTTCACCCTCAATCTGTTCATCGGTGTCATCATCGACAACTTCAACGAGCAGAAGAAGAAGGCCGGCGGCTCGCTAGAAATG

>GRCH02 Greece Chania

CAGGTGGGCAAGCAGCCGATTCGCGAGACCAACATCTACATGTACCTCTACTTTGTGTTCTTCATCATCTTCGGGTCGTTCTTCACCCTCAATCTGTTCATCGGTGTCATCATCGACAACTTCAACGAGCAGAAGAAGAAGGCCGGCGGCTCGCTAGAAATG

>GRCH03 Greece Chania

CAGGTGGGCAAGCAGCCGATTCGCGAGACCAACATCTACATGTACCTCTACTTTGTGTTCTTCATCATCTTCGGGTCGTTCTTCACCCTCAATCTGTTCATCGGTGTCATCATCGACAACTTCAACGAGCAGAAGAAGAAGGCCGGCGGCTCGCTAGAAATG

>GRCH04 Greece Chania

CAGGTGGGCAAGCAGCCGATTCGCGAGACCAACATCTACATGTACCTCTACTTTGTGTTCTTCATCATCTTCGGGTCGTTCTTCACCCTCAATCTGTTCATCGGTGTCATCATCGACAACTTCAACGAGCAGAAGAAGAAGGCCGGCGGCTCGCTAGAAATG

>GRCN02 Greece Chania

CAGGTGGGCAAGCAGCCGATTCGCGAGACCAACATCTACATGTACCTCTACTTCGTGTTCTTCATCATCTTCGGGTCGTTCTTCACCCTCAACCTGTTCATCGGTGTCATCATCGACAACTTCAACGAGCAGAAGAAGAAAGCCGGTGGCTCGCTGGAAATG

>GRCN03 Greece Chania

CAGGTGGGCAAGCAGCCGATYCGCGAGACCAACATCTACATGTACCTCTACTTYGTGTTCTTCATCATCTKCGGGTCGTTCTTCACCCTCAACCTGTTCATCGGTGTCATCATCGACAACTTCAACGAGCAGAAGAAGAAAGCCGGTGGCTCGCTGGAAATG

>GRCN04 Greece Chania

CAGGTGGGCAAGCAGCCAATTCGCGAGACCAACATCTACATGTACCTCTACTTCGTGTTCTTCATCATCTTCGGGTCGTTCTTCACCCTTAATCTGTTCATCGGTGTCATCATCGACAACTTCAACGAGCAGAAGAAGAAAGCCGGTGGCTCGCTGGAAATG

>GRCN05 Greece Chania

CAGGTGGGCAAGCAGCCRATYCGCGAGACCAACATCTACATGTACCTCTACTTYGTGTTCTTCATCATCTKCGGGTCGTTCTTCACCCTYAACCTGTTCATCGGTGTCATCATCGACAACTTCAACGAGCAGAAGAAGAAAGCCGGTGGCTCGCTGGAAATG

>GRCN07 Greece Chania

CAGGTGGGCAAGCAGCCGATYCGCGAGACCAACATCTACATGTACCTCTACTTYGTGTTCTTCATCATCTKCGGGTCGTTCTTCACCCTCAACCTGTTCATCGGTGTCATCATCGACAACTTCAACGAGCAGAAGAAGAAAGCCGGTGGCTCGCTGGAAATG

>GRCN09 Greece Chania

CAGGTGGGCAAGCAGCCRATYCGCGAGACCAACATCTACATGTACCTCTACTTYGTGTTCTTCATCATCTKCGGGTCGTTCTTCACCCTYAAYCTGTTCATCGGTGTCATCATCGACAACTTCAACGAGCAGAAGAAGAAAGCCGGTGGCTCGCTGGAAATG

>GRKA01 Greece Kavala

CAGGTGGGCAAGCAGCCGATTCGCGAGACCAACATCTACATGTACCTCTACTTCGTGTTCTTCATCATCTTCGGGTCGTTCTTCACCCTYAAYCTGTTCATCGGTGTCATCATCGACAACTTCAACGAGCAGAAGAAGAAAGCCGGTGGCTCGCTGGAAATG

>GRKA03 Greece Kavala

CAGGTGGGCAAGCAGCCRATYCGCGAGACCAACATCTACATGTACCTCTACTTYGTGTTCTTCATCATCTKCGGGTCGTTCTTCACCCTCAAYCTGTTCATCGGTGTCATCATCGACAACTTCAACGAGCAGAAGAAGAAAGCCGGTGGCTCGCTGGAAATG

>GRKA04 Greece Kavala

CAGGTGGGCAAGCAGCCRATYCGCGAGACCAACATCTACATGTACCTCTACTTYGTGTTCTTCATCATCTTCGGGTCGTTCTTCACCCTYAAYCTGTTCATCGGTGTCATCATCGACAACTTCAACGAGCAGAAGAAGAAAGCCGGTGGCTCGCTGGAAATG

>GRKA05 Greece Kavala

CAGGTGGGCAAGCAGCCGATYCGCGAGACCAACATCTACATGTACCTCTACTTYGTGTTCTTCATCATCTKCGGGTCGTTCTTCACCCTCAACCTGTTCATCGGTGTCATCATCGACAACTTCAACGAGCAGAAGAAGAAAGCCGGTGGCTCGCTGGAAATG

>ITRM06 Italy Roma

CAGGTGGGCAAGCAGCCGATTCGCGAGACCAACATCTACATGTACCTCTACTTCGTGTTCTTCATCATCTTCGGGTCGTTCTTCACCCTCAACCTGTTCATCGGTGTCATCATCGACAACTTCAACGAGCAGAAGAAGAAAGCCGGTGGCTCGCTGGAAATG

>ITRM12 Italy Roma

CAGGTGGGCAAGCAGCCRATTCGCGAGACCAACATCTACATGTACCTCTACTTCGTGTTCTTCATCATCTTCGGGTCGTTCTTCACCCTCAACCTGTTCATCGGTGTCATYATCGACAACTTCAACGAGCAGAAGAAGAAAGCCGGTGGCTCGCTGGAAATG

>ITRO08 Italy Roma

CAGGTGGGCAAGCAGCCGATTCGCGAGACCAACATCTACATGTACCTCTACTTCGTGTTCTTCAYCATCTTCGGGTCGTTCTTCACCCTYAAYCTGTTCATCGGTGTCATCATCGACAACTTCAACGAGCAGAAGAAGAAAGCCGGTGGCTCGCTGGAAATG

>ITRO10 Italy Roma

CAGGTGGGCAAGCAGCCGATTCGCGAGACCAACATCTACATGTACCTCTACTTCGTGTTCTTCATCATCTTCGGGTCGTTCTTCACCCTCAACCTGTTCATCGGTGTCATCATCGACAACTTCAACGAGCAGAAGAAGAAAGCCGGTGGCTCGCTGGAAATG

>ITRO11 Italy Roma

CAGGTGGGCAAGCAGCCGATTCGCGAGACCAACATCTACATGTACCTCTACTTCGTGTTCTTCATCATCTTCGGGTCGTTCTTCACCCTYAAYCTGTTCATCGGTGTCATCATCGACAACTTCAACGAGCAGAAGAAGAAAGCCGGTGGCTCGCTGGAAATG

>ITRO16 Italy Roma

CAGGTGGGCAAGCAGCCGATTCGCGAGACCAACATCTACATGTACCTCTACTTCGTGTTCTTCATCATCTTCGGGTCGTTCTTCACCCTCAACCTGTTCATCGGTGTCATCATCGACAACTTCAACGAGCAGAAGAAGAAAGCCGGTGGCTCGCTGGAAATG

>ITRO20 Italy Roma

CAGGTGGGCAAGCAGCCRATTCGCGAGACCAACATCTACATGTACCTCTACTTCGTGTTCTTCATCATCTTCGGGTCGTTCTTCACYCTCAACCTGTTCATCGGTGTCATYATCGACAACTTCAACGAGCAGAAGAAGAAAGCCGGTGGCTCGCTGGAAATG

>MTLU02 Malta Luqa

CAGGTGGGCAAGCAGCCRATTCGCGAGACCAACATCTACATGTACCTCTACTTCGTGTTCTTCATCATCTTCGGGTCGTTCTTCACCCTYAAYCTGTTCATCGGTGTCATCATCGACAACTTCAACGAGCAGAAGAAGAAAGCCGGTGGCTCGCTGGAAATG

>MTLU10 Malta Luqa

CAGGTGGGCAAGCAGCCGATTCGCGAGACCAACATCTACATGTACCTCTACTTCGTGTTCTTCATCATCTTCGGGTCGTTCTTCACCCTCAACCTGTTCATCGGTGTCATCATCGACAACTTCAACGAGCAGAAGAAGAAAGCCGGTGGCTCGCTGGAAATG

>MTLU15 Malta Luqa

------------------------------------CATGTACCTCTACTTCGTGTTCTTSATCATCTTCGGGTCGTTCTTCACCCTCAACCTGTTCATCGGTGTCATCATCGACAACTTCAACGAGCAGAAGAAGAAAGCCGGTGGCTCGCTGGAAATG

>MTLU16 Malta Luqa

CAGGTGGGCAAGCAGCCGATTCGCGAGACCAACATCTACATGTACCTCTACTTCGTGTTCTTCATCATCTTCGGGTCGTTCTTCACCCTCAACCTGTTCATCGGTGTCATCATCGACAACTTCAACGAGCAGAAGAAGAAAGCCGGTGGCTCGCTGGAAATG

>MTLU20 Malta Luqa

CAGGTGGGCAAGCAGCCGATTCGCGAGACCAACATCTACATGTACCTCTACTTCGTGTTCTTCATCATCTTCGGGTCGTTCTTCACCCTYAAYCTGTTCATCGGTGTCATCATCGACAACTTCAACGAGCAGAAGAAGAAAGCCGGTGGCTCGCTGGAAATG

>MTLU29 Malta Luqa

CAGGTGGGCAAGCAGCCAATTCGCGAGACCAACATCTACATGTACCTCTACTTYGTGTTCTTCATCATCTTCGGGTCGTTCTTCACCCTCAACCTGTTCATCGGTGTCATYATCGACAACTTCAACGAGCAGAAGAAGAAAGCCGGTGGCTCGCTGGAAATG

>MTLU32 Malta Luqa

CAGGTGGGCAAGCAGCCRATTCGCGAGACCAACATCTACATGTACCTCTACTTYGTGTTCTTCATCATCTTCGGGTCGTTCTTCACCCTCAACCTGTTCATCGGTGTCATCATCGACAACTTCAACGAGCAGAAGAAGAAAGCCGGTGGCTCGCTGGAAATG

>MTLU40 Malta Luqa

CAGGTGGGCAAGCAGCCGATTCGCGAGACCAACATCTACATGTACCTCTACTTCGTGTTCTTCATCATCTTCGGGTCGTTCTTCACCCTYAAYCTGTTCATCGGTGTCATCATCGACAACTTCAACGAGCAGAAGAAGAAAGCCGGTGGCTCGCTGGAAATG

>PTPN02 Portugal Penafiel

CAGGTGGGCAAGCAGCCAATTCGCGAGACCAACATCTACATGTACCTCTACTTCGTGTTCTTCATCATCTTCGGGTCGTTCTTCACCCTTAATCTGTTCATCGGTGTCATCATCGACAACTTCAACGAGCAGAAGAAGAAAGCCGGTGGCTCGCTGGAAATG

>PTPN09 Portugal Penafiel

CAGGTGGGCAAGCAGCCGATTCGCGAGACCAACATCTACATGTACCTCTACTTCGTGTTCTTCATCATCTTCGGGTCGTTCTTCACYCTYAAYCTGTTCATCGGTGTCATCATCGACAACTTCAACGAGCAGAAGAAGAAAGCCGGTGGCTCGCTGGAAATG

>PTPN10 Portugal Penafiel

CAGGTGGGCAAGCAGCCGATTCGCGAGACCAACATCTACATGTACCTCTACTTCGTGTTCTTCATCATCTTCGGGTCGTTCTTCACCCTCAACCTGTTCATCGGTGTCATCATCGACAACTTCAACGAGCAGAAGAAGAAAGCCGGTGGCTCGCTGGAAATG

>PTPN12 Portugal Penafiel

CAGGTGGGCAAGCAGCCGATTCGCGGA-CCAACATCTACATGTACCTCTACTTCGTGTTCTTCATCATCTTCGGGTCGTTCTTCACCCTCAACCTGTTCATCGGTGTCATCATCGACAACTTCAACGAGCAGAAGAAGAAAGCCGGTGGCTCGCTGGAAATG

>PTQT02 Portugal Loul

-AGGTGGGCAAGCAGCCRATTCGCGAGACCAACATCTACATGTACCTCTACTTCGTGTTCTTCATCATCTTCGGGTCGTTCTTCACTGTCAACCTGTTCATCGGWGTCATCATCGACAACTTCAACGAGCAGAAGAAGAAAGCCGGTGGCTCGCTGGAAATG

>PTQT16 Portugal Loul

CAGGTGGGCAAGCAGCCRATTCGCGAGACCAACATCTACATGTACCTCTACTTCGTGTTCTTCATCATCTTCGGGTCGTTCTTCACCCTYAAYCTGTTCATCGGTGTCATCATCGACAACTTCAACGAGCAGAAGAAGAAAGCCGGTGGCTCGCTGGAAATG

>ROBU12 Romania Bucharest

CAGGTGGGCAAGCAGCCGATTCGCGAGWCCAACATCTACATGTACCTCTACTTCGTGTTCTTCATCATCTTCGGGTCGTTCTTCACCCTCAACCTGTTCATCGGTGTCAT----------------------------------------------------

>ROBU19 Romania Bucharest

---------------CCGATTCGCGAGACCAACATCTACATGTACCTCTACTTCGTGTTCTTCATCATCTTCGGGTCGTTCTTCACCCTCAACCTGTTCATCGGTGTCATCATCGACAACTTCAACGAGCAGA-----------------------------

>ROPL20 Romania Plopsoru

CAGGTGGGCAAGCAGCCGATTCGCGAGACCAACATCTACATGTACCTCTACTTTGTGTTCTTCATCATCTTCGGGTCGTTCTTCACGCTCAACCTGTTCATCGGTGTCATCATCGACAACTTCAACGAGCAGAAGAAGAAAGCCGGTGGCTCGCTGGAAATG

>ROPL26 Romania Plopsoru

CAGGTGGGCAAGCAGCCGATTCGCGAGACCAACATCTACATGTACCTCTACTTCGTGTTCTTCATCATCTTCGGGTCGTTCTTCACCCTCAACCTGTTCAT-------------------------------------------------------------

>ROSM15 Romania Satu Mare

-------------------------------------MCATGTRCCTCTACTTCGTGTTCTTCATCATCTTCGGGTCGTTCTTCACCCTCAACCTGTTCATCGGTGTCATCATCGACAACTTCAACGAGCAGAAGAAGAAAGCCGGTGGCTCGCTGGAAATG

>ROSM16 Romania Satu Mare

CAGGTGGGCAAGCAGCCGATTCGCGAGACCAACATCTACATGTACCTCTACTTYGTGTTCTTCAYCATCTTCGGGTCGTTCTTCACCCTYAAYCTGTTCATCGGTGTCATCATCGACAACTTCAACGAGCAGAAGAAGAAAGCCGGTGGCTCGCTGGAAATG

>RUBE04 Russia Beloretchensk

CAGGTGGGCAAGCAGCCGATTCGCGAGACCAACATCTACATGTACCTCTACTTCGTGTTCTTCATCATCTTCGGGTCGTTCTTCACCCTYAAYCTGTTCATCGGTGTCATCATCGACAACTTCAACGAGCAGAAGAAGAAAGCCGGTGGCTCGCTGGAAATG

>RUBE20 Russia Beloretchensk

CAGGTGGGCAAGCAGCCGATTCGCGAGACCAACATCTACATGTACCTCTACTTCGTGTTCTTCATCATCTTCGGGTCGTTCTTCACCCTCAACCTGTTCATCGGTGTCATCATCGACAACTTCAACGAGCAGAAGAAGAAAGCCGGTGGCTCGCTGGAAATG

>RUPL02 Russia Plastounka

CAGGTGGGCAAGCAGCCGATTCGCGAGACCAACATCTACATGTACCTCTACTTCGTGTTCTTCATCATCTTCGGGTCGTTCTTCACCCTCAACCTGTTCATCGGTGTCATCATCGACAACTTCAACGAGCAGAAGAAGAAAGCCGGTGGCTCGCTGGAAATG

>RUPL10 Russia Plastounka

CAGGTGGGCAAGCAGCCGATTCGCGAGACCAACATCTACATGTACCTCTACTTCGTGTTCTTCATCATCTTCGGGTCGTTCTTCACCCTCAACCTGTACATCGGTGTCATCATCGACAACTTCAACGAGCAGAAGAAGAAAGCCGGTGGCTCGCTGGAAATG

>RURM04 Russia Russkaya Mamayka

CAGGTGGGCAAGCAGCCGATTCGCGAGWCCAACATCTACATGTACCTCTACTTCGTGTTCTTCATCATCTTCGGGTCGTTCTTCACCCTTAATCTGTTCATCGGTGTCATCATCGACAACTTCAACGAGCAGAAGAAGAAAGCC------------------

>RUSO02 Russia Sochi

CAGGTGGGCAAGCAGCCGATTCGCGAGACCAACATCTACATGTACCTCTACTTCGTGTTCTTCATCATCTTCGGGTCGTTCTTCACCCTCAACCTGTTCATCGGTGTCATCATCGACAACTTCAACGAGCAGAAGAAGAAAGCCGGTGGCTCGCTGGAAATG

>SLGO01 Slovenia Ajdov ?ina

CAGGTGGGCAAGCAGCCRATTCGCGAGACCAACATCTACATGTACCTCTACTTCGTGTTCTTCATCATCTTCGGGTCGTTCTTCACCCTYAAYCTGTTCATCGGTGTCATCATCGACAACTTCAACGAGCAGAAGAAGAAAGCCGGTGGCTCGCTGGAAATG

>SLGO02 Slovenia Ajdov ?ina

CAGGTGGGCAAGCAGCCAATTCGCGAGACCAACATCTACATGTACCTCTACTTCGTGTTCTTCATCATCTTCGGGTCGTTCTTCACCCTTAATCTGTTCATCGGTGTCATCATCGACAACTTCAACGAGCAGAAGAAGAAAGCCGGTGGCTCGCTGGAAATG

>SLGO03 Slovenia Ajdov ?ina

CAGGTGGGCAAGCAGCCRATTCGCGAGACCAACATCTACATGTACCTCTACTTCGTGTTCTTCATCATCTTCGGGTCGTTCTTCACCCTCAACCTGTTCATCGGTGTCATCATCGACAACTTCAACGAGCAGAAGAAGAAAGCCGGTGGCTCGCTGGAAATG

>SLGO05 Slovenia Ajdov ?ina

----------------------------------------------------TCGTGTTCTTCATCATCTTCGGGTCGTTCTTCACCCTCAACCTGTTCATCGGTGTCATCATCGACAACTTCAACGAGCAGAAGAAGAAAGCCGGTGGCTCGCTGGAAATG

>SLGO14 Slovenia Ajdov ?ina

CAGGTGGGCAAGCAGCCGATTCGCGAGACCAACATCTACATGTACCTCTACTTCGTGTTCTTCATCATCTTCGGGTCGTTCTTCACCCTCAACCTGTTCATCGGTGTCATCATCGACAACTTCAACGAGCAGAAGAAGAAAGCCGGTGGCTCGCTGGAAATG

>SLGO18 Slovenia Ajdov ?ina

CAGGTGGGCAAGCAGCCAATTCGCGAGACCAACATCTACATGTACCTCTACTTCGTGTTCTTCATCATCTTCGGGTCGTTCTTCACCCTCAACCTGTTCATCGGTGTCATCATCGACAACTTCAACGAGCAGAAGAAGAAAGCCGGTGGCTCGCTGGAAATG

>SLGO38 Slovenia Ajdov ?ina

CAGGTGGGCAAGCAGCCGATTCGCGAGACCAACATCTACATGTACCTCTACTTCGTGTTCTTCATCATCTTCGGGTCGTTCTTCACCCTCAACCTGTTCATCGGTGTCATCATCGACAACTTCAACGAGCAGAAGAAGAAAGCCGGTGGCTCGCTGGAAATG

>SLGO39 Slovenia Ajdov ?ina

CAGGTGGGCAAGCAGCCRATTCGCGAGACCAACATCTACATGTACCTCTACTTCGTGTTCTTCATCATCTTCGGGTCGTTCTTCACCCTCAACCTGTTCATCGGTGTCATCATCGACAACTTCAACGAGCAGAAGAAGAAAGCCGGTGGCTCGCTGGAAATG

>SRAP02 Serbia Apatin

CAGGTGGGCAAGCAGCCRATTCGCGAGACCAACATCTACATGTACCTCTACTTCGTGTTCTTCATCATCTTCGGGTCGTTCTTCACCCTYAAYCTGTTCATCGGTGTCATCATCGACAACTTCAACGAGCAGAAGAAGAAAGCCGGTGGCTCGCTGGAAATG

>SRAP22 Serbia Apatin

CAGGTGGGCAAGCAGCCRATTCGCGAGACCAACATCTACATGTACCTCTACTTCGTGTTCTTCATCATCTTCGGGTCGTTCTTCACCCTYAAYCTGTTCATCGGTGTCATCATCGACAACTTCAACGAGCAGAAGAAGAAAGCCGGTGGCTCGCTGGAAATG

>SRAP40 Serbia Apatin

CAGGTGGGCAAGCAGCCRATTCGCGAGACCAACATCTACATGTACCTCTACTTCGTGTTCTTCATCATCTTCGGGTCGTTCTTCACCCTYAAYCTGTTCATCGGTGTCATCATCGACAACTTCAACGAGCAGAAGAAGAAAGCCGGTGGCTCGCTGGAAATG

>TRGN08 Turkey Igneada

CAGGTGGGCAAGCAGCCGATTCGCGAGACCAACATCTACATGTACCTCTACTTCGTGTTCTTCATCATCTTCGGGTCGTTCTTCACCCTCAACCTGTTCATCGGTGTCATCATCGACAACTTCAACGAGCAGAAGAAGAAAGCCGGTGGCTCGCTGGAAATG

>TRGN09 Turkey Igneada

CAGGTGGGCAAGCAGCCGATTCGCGAGACCAACATCTACATGTACCTCTACTTCGTGTTCTTCAYCATCTTCGGGTCGTTCTTCACCCTYAAYCTGTTCATCGGTGTCATCATCGACAACTTCAACGAGCAGAAGAAGAAAGCCGGTGGCTCGCTGGAAATG

>TRGN12 Turkey Igneada

CAGGTGGGCAAGCAGCCGATTCGCGAGACCAACATCTACATGTACCTCTACTTYGTGTTCTTCATCATCTTCGGGTCGTTCTTCACCCTCAACCTGTTCATCGGTGTCATCATCGACAACTTCAACGAGCAGAAGAAGAAAGCCGGTGGCTCGCTGGAAATG

>TRGN21 Turkey Igneada

CAGGTGGGCAAGCAGCCGATTCGCGAGACCAACATCTACATGTACCTCTACTTCGTGTTCTTCATCATCTTCGGGTCGTTCTTCACCCTCAACCTGTTCATCGGTGTCATCATCGACAACTTCAACGAGCAGAAGAAGAAAGCCGGTGGCTCGCTGGAAATG

>TRGN22 Turkey Igneada

CAGGTGGGCAAGCAGCCGATTCGCGAGACCAACATCTACATGTACCTCTACTTCGTGTTCTTCAYCATCTTCGGGTCGTTCTTCACCCTYAAYCTGTTCATCGGTGTCATCATCGACAACTTCAACGAGCAGAAGAAGAAAGCCGGTGGCTCGCTGGAAATG

>TRIS03 Turkey Istanbul

CAGGTGGGCAAGCAGCCRATTCGCGAGACCAACATCTACATGTACCTCTACTTCGTGTTCTTCATCATCTTCGGGTCGTTCTTCACCCTYAAYCTGTTCATCGGTGTCATCATCGACAACTTCAACGAGCAGAAGAAGAAAGCCGGTGGCTCGCTGGAAATG

>TRIS06 Turkey Istanbul

CAGGTGGGCAAGCAGCCGATTCGCGAGACCAACATCTACATGTACCTCTACTTCGTGTTCTTCATCATCTTCGGGTCGTTCTTCACCCTCAACCTGTTCATCGGTGTCATCATCGACAACTTCAACGAGCAGAAGAAGAAAGCCGGTGGCTCGCTGGAAATG

>TRIS14 Turkey Istanbul

CAGGTGGGCAAGCAGCCGATTCGCGAGACCAACATCTACATGTACCTCTACTTCGTGTTCTTCATCATCTTCGGGTCGTTCTTCACYCTCAACCTGTTCATCGGTGTCATCATCGACAACTTCAACGAGCAGAAGAAGAAAGCCGGTGGCTCGCTGGAAATG

>TRIS16 Turkey Istanbul

CAGGTGGGCAAGCAGCCGATTCGCGAGACCAACATCTACATGTACCTCTACTTCGTGTTCTTCATCATCTTCGGGTCGTTCTTCACYCTCAACCTGTTCATCGGTGTCATCATCGACAACTTCAACGAGCAGAAGAAGAAAGCCGGTGGCTCGCTGGAAATG

>TRIS17 Turkey Istanbul

CAGGTGGGCAAGCAGCCRATTCGCGAGACCAACATCTACATGTACCTCTACTTCGTGTTCTTCATCATCTTCGGGTCGTTCTTCACCCTYAAYCTGTTCATCGGTGTCATCATCGACAACTTCAACGAGCAGAAGAAGAAAGCCGGTGGCTCGCTGGAAATG

>TRIS23 Turkey Istanbul

CAGGTGGGCAAGCAGCCGATTCGCGAGACCAACATCTACATGTACCTCTACTTCGTGTTCTTCATCATCTTCGGGTCGTTCTTCACCCTCAACCTGTTCATCGGTGTCATCATCGACAACTTCAACGAGCAGAAGAAGAAAGCCGGTGGCTCGCTGGAAATG

>TRIS29 Turkey Istanbul

CAGGTGGGCAAGCAGCCRATTCGCGAGACCAACATCTACATGTACCTCTACTTCGTGTTCTTCATCATCTTCGGGTCGTTCTTCACCCTYAAYCTGTTCATCGGTGTCATCATCGACAACTTCAACGAGCAGAAGAAGAAAGCCGGTGGCTCGCTGGAAATG

>TRIS34 Turkey Istanbul

CAGGTGGGCAAGCAGCCGATTCGCGAGACCAACATCTACATGTACCTCTACTTCGTGTTCTTCATCATCTTCGGGTCGTTCTTCACCCTCAACCTGTTCATCGGTGTCATCATCGACAACTTCAACGAGCAGAAGAAGAAAGCCGGTGGCTCGCTGGAAATG

>TRIS36 Turkey Istanbul

CAGGTGGGCAAGCAGCCGATTCGCGAGACCAACATCTACATGTACCTCTACTTCGTGTTCTTCAYCATCTTCGGGTCGTTCTTCACCCTYAAYCTGTTCATCGGTGTCATCATCGACAACTTCAACGAGCAGAAGAAGAAAGCCGGTGGCTCGCTGGAAATG

>TRIS40 Turkey Istanbul

CAGGTGGGCAAGCAGCCGATTCGCGAGACCAACATCTACATGTACCTCTACTTCGTGTTCTTCATCATCTTCGGGTCGTTCTTCACTCTCAACCTGTTCATCGGTGTCATCATCGACAACTTCAACGAGCAGAAGAAGAAAGCCGGTGGCTCGCTGGAAATG

>TRIS44 Turkey Istanbul

CAGGTGGGCAAGCAGCCGATTCGCGAGACCAACATCTACATGTACCTCTACTTCGTGTTCTTCATCATCTTCGGGTCGTTCTTCACCCTCAACCTGTTCATCGGTGTCATCATCGACAACTTCAACGAGCAGAAGAAGAAAGCCGGTGGCTCGCTGGAAATG

>TRLG04 Turkey Aliaga

CAGGTGGGCAAGCAGCCRATTCGCGAGACCAACATCTACATGTACCTCTACTTCGTGTTCTTCATCATCTTCGGGTCGTTCTTCACKCTCAACCTGTTCATCGGTGTCATCATCGACAACTTCAACGAGCAGAAGAAGAAAGCCGGTGGCTCGCTGGAAATG

>TRLG05 Turkey Aliaga

CAGGTGGGCAAGCAGCCAATTCGCGAGACCAACATCTACATGTACCTCTACTTCGTGTTCTTCATCATCTTCGGGTCGTTCTTCACCCTTAATCTGTTCATCGGTGTCATCATCGACAACTTCAACGAGCAGAAGAAGAAAGCCGGTGGCTCGCTGGAAATG

>TRLG12 Turkey Aliaga

CAGGTGGGCAAGCAGCCAATTCGCGAGACCAACATCTACATGTACCTCTACTTCGTGTTCTTCATCATCTTCGGGTCGTTCTTCACSCTYAAYCTGTTCATCGGTGTCATCATCGACAACTTCAACGAGCAGAAGAAGAAAGCCGGTGGCTCGCTGGAAATG

>TRLG17 Turkey Aliaga

CAGGTGGGCAAGCAGCCRATTCGCGAGACCAACATCTACATGTACCTCTACTTCGTGTTCTTCATCATCTTCGGGTCGTTCTTCACCCTYAAYCTGTTCATCGGTGTCATCATCGACAACTTCAACGAGCAGAAGAAGAAAGCCGGTGGCTCGCTGGAAATG

>TRLG41 Turkey Aliaga

-----------------------------CAACATCTACATGTACCTCTACTTCGTGTTCTTCATCATCTTCGGGTCGTTCTTCACCCTCAACCTGTTCATCGGTGTCATCATCGACAACTTCAACGAGCAGAAGAAGAAAGCCGGTGGCTCGCTGGAAATG

>TRTR22 Turkey Trabzon

CAGGTGGGCAAGCAGCCAATTCGCGAGACCAACATCTACATGTACCTCTACTTCGTGTTCTTCATCATCTTCGGGTCGTTCTTCACGCTCAACCTGTTCATCGGTGTCATCATCGACAACTTCAACGAGCAGAAGAAGAAAGCCGGTGGCTCGCTGGAAATG

>ITBR04 Italy Bari

CAGGTGGGCAAGCAGCCGATTCGCGAGACCAACATCTACATGTACCTCTACTTCGTGTTCTTCATCATCTTCGGGTCGTTCTTCACCCTCAACCTGTTCATCGGTGTCATCATCGACAACTTCAACGAGCAGAAGAAGAAAGCCGGTGGCTCGCTGGAAATG

>ITBL03 Italy Bologna

CAGGTGGGCAAGCAGCCGATTCGCGAGACCAACATCTACATGTACCTCTACTTCGTGTTCTTCACCATCTTCGGGTCGTTCTTCACCCTTAATCTGTTCATCGGTGTCATCATCGACAACTTCAACGAGCAGAAGAAGAAAGCCGGTGGCTCGCTGGAAATG

>ITBO05 Italy Bologna

CAGGTGGGCAAGCAGCCRATTCGCGAGACCAACATCTACATGTACCTCTACTTCGTGTTCTTCATCATCTTCGGGTCGTTCTTCACCCTYAAYCTGTTCATCGGTGTCATCATCGACAACTTCAACGAGCAGAAGAAGAAAGCCGGTGGCTCGCTGGAAATG

>ITBO04 Italy Bologna

CAGGTGGGCAAGCAGCCGATTCGCGAGACCAACATCTACATGTACCTCTACTTCGTGTTCTTCATCATCTTCGGGTCGTTCTTCACCCTCAACCTGTTCATCGGTGTCATCATCGACAACTTCAACGAGCAGAAGAAGAAAGCCGGTGGCTCGCTGGAAATG

>ITVL02 Italy Bari

CAGGTGGGCAAGCAGCCGATTCGCGAGACCAACATCTACATGTACCTCTACTTCGTGTTCTTCAYCATCTTCGGGTCGTTCTTCACCCTYAAYCTGTTCATCGGTGTCATCATCGACAACTTCAACGAGCAGAAGAAGAAAGCCGGTGGCTCGCTGGAAATG

>ITVL05 Italy Bari

CAGGTGGGCAAGCAGCCGATTCGCGAGACCAACATCTACATGTACCTCTACTTCGTGTTCTTCATCATCTTCGGGTCGTTCTTCACCCTCAACCTGTTCATCGGTGTCATCATCGACAACTTCAACGAGCAGAAGAAGAAAGCCGGTGGCTCGCTGGAAATG

>ITFI05 Italy Gemona del Friuli

CAGGTGGGCAAGCAGCCGATTCGCGAGACCAACATCTACATGTACCTCTACTTSGTGTTCTTCATCATCTTCGGGTCGTTCTTCACCCTCAACCTGTTCATCGGTGTCATCATCGACAACTTCAACGAGCAGAAGAAGAAAGCCGGTGGCTCGCTGGAAATG

>ITFR03 Italy Gemona del Friuli

CAGGTGGGCAAGCAGCCGATTCGCGAGACCAACATCTACATGTACCTCTACTTCGTGTTCTTCATCATCTTCGGGTCGTTCTTCACCCTCAACCTGTTCATCGGTGTCATCATCGACAACTTCAACGAGCAGAAGAAGAAAGCCGGTGGCTCGCTGGAAATG

>ITVL04 Italy Bari

CAGGTGGGCAAGCAGCCGATTCGCGAGACCAACATCTACATGTACCTCTACTTCGTGTTCTTCATCATCTTCGGGTCGTTCTTCACCCTCAACCTGTTCATCGGTGTCATCATCGACAACTTCAACGAGCAGAAGAAGAAAGCCGGTGGCTCGCTGGAAATG

>ITFR02 Italy Gemona del Friuli

CAGGTGGGCAAGCAGCCGATTCGCGAGACCAACATCTACATGTACCTCTACTTCGTGTTCTTCATCATCTTCGGGTCGTTCTTCACYCTCAACCTGTTCATCGGTGTCATCATCGACAACTTCAACGAGCAGAAGAAGAAAGCCGGTGGCTCGCTGGAAATG

>ITBO02 Italy Bologna

CAGGTGGGCAAGCAGCCGATTCGCGAGACCAACATCTACATGTACCTCTACTTCGTGTTCTTCATCATCTTCGGGTCGTTCTTCACCCTCAACCTGTTCATCGGTGTCATCATCGACAACTTCAACGAGCAGAAGAAGAAAGCCGGTGGCTCGCTGGAAATG

>ITBO03 Italy Bologna

CAGGTGGGCAAGCAGCCGATTCGCGAGACCAACATCTACATGTACCTCTACTTYGTGTTCTTCATCATCTTCGGGTCGTTCTTCACSCTYAAYCTGTTCATCGGTGTCATCATCGACAACTTCAACGAGCAGAAGAAGAAAGCCGGTGGCTCGCTGGAAATG

>ITVL03 Italy Bari

CAGGTGGGCAAGCAGCCGATTCGCGAGACCAACATCTACATGTACCTCTACTTCGTGTTCTTCATCATCTTCGGGTCGTTCTTCACCCTCAACCTGTTCATCGGTGTCATCATCGACAACTTCAACGAGCAGAAGAAGAAAGCCGGTGGCTCGCTGGAAATG

>ITBL01 Italy Bologna

CAGGTGGGCAAGCAGCCRATTCGCGAGACCAACATCTACATGTACCTCTACTTCGTGTTCTTCATCATCTTCGGGTCGTTCTTCACCCTCAACCTGTTCATCGGTGTCATYATCGACAACTTCAACGAGCAGAAGAAGAAAGCCGGTGGCTCGCTGGAAATG

>ITFI01 Italy Gemona del Friuli

CAGGTGGGCAAGCAGCCRATTCGCGAGACCAACATCTACATGTACCTCTACTTCGTGTTCTTCATCATCTTCGGGTCGTTCTTCACCCTCAACCTGTTCATCGGTGTCATCATCGACAACTTCAACGAGCAGAAGAAGAAAGCCGGTGGCTCGCTGGAAATG

>ITBL02 Italy Bologna

CAGGTGGGCAAGCAGCCGATTCGCGAGACCAACATCTACATGTACCTCTACTTCGTGTTCTTCACCATCTTCGGGTCGTTCTTCACCCTTAATCTGTTCATCGGTGTCATCATCGACAACTTCAACGAGCAGAAGAAGAAAGCCGGTGGCTCGCTGGAAATG

>ITBL05 Italy Bologna

CAGGTGGGCAAGCAGCCGATTCGCGAGACCAACATCTACATGTACCTCTACTTCGTGTTCTTCAYCATCTTCGGGYCGTTCTTCACCCTYAAYCTGTTCATCGGTGTCATCATCGACAACTTCAACGAGCAGAAGAAGAAAGCCGGTGGCTCGCTGGAAATG

>ITFI04 Italy Gemona del Friuli

CAGGTGGGCAAGCAGCCGATTCGCGAGACCAACATCTACATGTACCTCTATTTCGTGTTCTTCATCATCTTCGGGTCGTTCTTCACCCTCAACCTGTTCATCGGTGTCATCATCGACAACTTCAACGAGCAGAAGAAGAAAGCCGGTGGCTCGCTGGAAATG

>ITBL04 Italy Bologna

CAGGTGGGCAAGCAGCCRATTCGCGAGACCAACATCTACATGTACCTCTACTTCGTGTTCTTCATCATCTTCGGGTCGTTCTTCACCCTYAAYCTGTTCATCGGTGTCATCATCGACAACTTCAACGAGCAGAAGAAGAAAGCCGGTGGCTCGCTGGAAATG

>ITBO01 Italy Bologna

CAGGTGGGCAAGCAGCCGATTCGCGAGACCAACATCTACATGTACCTCTACTTYGTGTTCTTCATCATCTTCGGGTCGTTCTTCACSCTYAAYCTGTTCATCGGTGTCATCATCGACAACTTCAACGAGCAGAAGAAGAAAGCCGGTGGCTCGCTGGAAATG

>ITFR05 Italy Gemona del Friuli

CAGGTGGGCAAGCAGCCAATTCGCGAGACCAACATCTACATGTACCTCTACTTCGTGTTCTTCATCATCTTCGGGTCGTTCTTCACCCTYAAYCTGTTCATCGGTGTCATCATCGACAACTTCAACGAGCAGAAGAAGAAAGCCGGTGGCTCGCTGGAAATG

>ITFR04 Italy Gemona del Friuli

CAGGTGGGCAAGCAGCCGATTCGCGAGACCAACATCTACATGTACCTCTACTTYGTGTTCTTCATCATCTTCGGGTCGTTCTTCACCCTCAACCTGTTCATCGGTGTCATCATCGACAACTTCAACGAGCAGAAGAAGAAAGCCGGTGGCTCGCTGGAAATG

>ITVL01 Italy Bari

CAGGTGGGCAAGCAGCCGATTCGCGAGACCAACATCTACATGTACCTCTACTTCGTGTTCTTCATCATCTTCGGGTCGTTCTTCACCCTCAACCTGTTCATCGGTGTCATCATCGACAACTTCAACGAGCAGAAGAAGAAAGCCGGTGGCTCGCTGGAAATG

>19ME370 Montenegro Podgorica

CAGGTGGGCAAGCAGCCAATTCGCGAGACCAACATCTACATGTACCTCTACTTCGTGTTCTTCATCATCTTCGGGTCGTTCTTCACCCTCAACCTGTTCATCGGTGTCATCATCGACAACTTCAACGAGCAGAAGAAGAAAGCCGGTGGCTCGCTGGAAATG

>ITRM05 Italy Roma

CAGGTGGGCAAGCAGCCGATTCGCGAGACCAACATCTACATGTACCTCTACTTCGTGTTCTTCATCATCTTYGGGTCGTTCTTCACCCTCAACCTGTTCATCGGTGTCATCATCGACAACTTCAACGAGCAGAAGAAGAAAGCCGGTGGCTCGCTGGAAATG

>GRCC02 Greece Chania

CAGGTGGGCAAGCAGCCGATTCGCGAGACCAACATCTACATGTACCTCTACTTCGTGTTCTTCATCATCTTCGGGTCGTTCTTCACCCTCAACCTGTTCATCGGTGTCATCATCGACAACTTCAACGAGCAGAAGAAGAAAGCCGGTGGCTCGCTGGAAATG

>20FR013 France Nice

CAGGTGGGCAAGCAGCCGATTCGCGAGACCAACATCTACATGTACCTCTACTTCGTGTTCTTCAYCATCTTCGGGTCGTTCTTCACCCTYAAYCTGTTCATCGGTGTCATCATCGACAACTTCAACGAGCAGAAGAAGAAAGCCGGTGGCTCGCTGGAAATG

>ITRM04 Italy Roma

CAGGTGGGCAAGCAGCCRATTCGCGAGACCAACATCTACATGTACCTCTACTTCGTGTTCTTCATCATCTTCGGGTCGTTCTTCACCCTYAAYCTGTTCATCGGTGTCATCATCGACAACTTCAACGAGCAGAAGAAGAAAGCCGGTGGCTCGCTGGAAATG

>BG20190014 Bulgaria Burgas

CAGGTGGGCAAGCAGCCRATTCGCGAGACCAACATCTACATGTACCTCTACTTCGTGTTCTTCATCATCTTCGGGTCGTTCTTCACCCTCAACCTGTTCATCGGTGTCATYATCGACAACTTCAACGWGCAGAAGAAGAAASCCGGTGGCTCKCTGGAAATG

>ROBU14 Romania Bucharest

CAGGTGGGCAAGCAGCCGATYCGCGAGACCAACATCTACATGTACCTCTACTTYGTGTTCTTCATCATCTKCGGGTCGTTCTTCACCCTCAACCTGTTCATCGGTGTCATCATCGACAACTTCAACGAGCAGAAGAAGAAAGCCGGTGGCTCGCTGGAAATG

>ITRM03 Italy Roma

CAGGTGGGCAAGCAGCCRATTCGCGAGACCAACATCTACATGTACCTCTACTTCGTGTTCTTCATCATCTTCGGGTCGTTCTTCACCCTYAAYCTGTTCATCGGTGTCATCATCGACAACTTCAACGAGCAGAAGAAGAAAGCCGGTGGCTCGCTGGAAATG

>ITRM02 Italy Roma

CAGGTGGGCAAGCAGCCGATTCGCGAGACCAACATCTACATGTACCTCTACTTCGTGTTCTTCATCATCTTYGGGTCGTTCTTCACCCTCAACCTGTTCATCGGTGTCATCATCGACAACTTCAACGAGCAGAAGAAGAAAGCCGGTGGCTCGCTGGAAATG

>ITRM01 Italy Roma

CAGGTGGGCAAGCAGCCRATTCGCGAGACCAACATCTACATGTACCTCTACTTCGTGTTCTTCATCATCTTCGGGTSRTTCTTCRCCCTYAAYCTGTTCATCGGTGTCATCATCGACAACTTCAACGAGCAGAAGAAGAAAGCCGGTGGCTCGCTGGAAATG

>GRAE10 Greece Athens(AIA)

CAGGTGGGCAAGCAGCCGATCCGCGAGACCAACATCTACATGTACCTCTACTTTGTGTTCTTCATCATCTGCGGGTCGTTCTTCACCCTCAACCTGTTCATCGGTGTCATCATCGACAACTTCAACGAGCAGAAGAAGAAAGCCGGTGGCTCGCTGGAAATG

>GRKB05 Greece Kavala

CAGGTGGGCAAGCAGCCGATTCGCGAGACCAACATCTACATGTACCTCTACTTYGTGTTCTTCATCATCTTCGGGTCGTTCTTCACCCTCAACCTGTTCATCGGTGTCATCATCGACAACTTCAACGAGCAGAAGAAGAAAGCCGGTGGCTCGCTGGAAATG

>GRAE08 Greece Athens(AIA)

CAGGTGGGCAAGCAGCCGATCCGCGAGACCAACATCTACATGTACCTCTACTTTGTGTTCTTCATCATCTGCGGGTCGTTCTTCACCCTCAACCTGTTCATCGGTGTCATCATCGACAACTTCAACGAGCAGAAGAAGAAAGCCGGTGGCTCGCTGGAAATG

>GRKB02 Greece Kavala

CAGGTGGGCAAGCAGCCGATTCGCGAGACCAACATCTACATGTACCTCTACTTCGTGTTCTTCATCATCTTCGGGTCGTTCTTCACCCTCAACCTGTTCATCGGTGTCATCATCGACAACTTCAACGAGCAGAAGAAGAAAGCCGGTGGCTCGCTGGAAATG

>GRAE06 Greece Athens(AIA)

CAGGTGGGCAAGCAGCCGATYCGCGAGACCAACATCTACATGTACCTCTACTTYGTGTTCTTCATCATCTKCGGGTCGTTCTTCACCCTYAAYCTGTTCATCGGTGTCATCATCGACAACTTCAACGAGCAGAAGAAGAAAGCCGGTGGCTCGCTGGAAAG-

>20CH044 Switzerland Basel

CAGGTGGGCAAGCAGCCGATTCGCGAGACCAACATCTACATGTACCTCTACTTYGTGTTCTTCATCATCTTCGGGTCGTTCTTCACCCTYAAYCTGTTCATCGGTGTCATCATCGACAACTTCAACGAGCAGAAGAAGAAAGCCGGTGGCTCGCTGGAAATG

>GRAE04 Greece Athens(AIA)

CAGGTGGGCAAGCAGCCGATCCGCGAGACCAACATCTACATGTACCTCTACTTTGTGTTCTTCATCATCTGCGGGTCGTTCTTCACCCTCAACCTGTTCATCGGTGTCATCATCGACAACTTCAACGAGCAGAAGAAGAAAGCCGGTGGCTCGCTGGAAATG

>19ME367 Montenegro Podgorica

CAGGTGGGCAAGCAGCCRATTCGCGAGACCAACATCTACATGTACCTCTACTTCGTGTTCTTCATCATCTTCGGGTCGTTCTTCACCCTYAAYCTGTTCATCGGTGTCATCATCGACAACTTCAACGAGCAGAWGAAGAAWGCCGGTGGCTCGCTGGAAATG

>GRCA02 Greece Chania

CAGGTGGGCAAGCAGCCGATCCGCGAGACCAACATCTACATGTACCTCTACTTTGTGTTCTTCATCATCTGCGGGTCGTTCTTCACCCTCAACCTGTTCATCGGTGTCATCATCGACAACTTCAACGAGCAGAAGAAGAAAGCCGGTGGCTCGCTGGAAATG

>20FR012 France Nice

CAGGTGGGCAAGCAGCCRATTCGCGAGACCAACATCTACATGTACCTCTACTTCGTGTTCTTCATCATCTTCGGGTCGTTCTTCACCCTYAAYCTGTTCATCGGTGTCATCATCGACAACTTCAACGAGCAGAAGAAGAAAGCCGGTGGCTCGCTGGAAATG

>BG20190011 Bulgaria Burgas

CAGGTGGGCAAGCAGCCGATTCGCGAGACCAACATCTACATGTACCTCTACTTCGTGTTCTTCATCATCTTCGGGTCGTTCTTCACCCTCAACCTGTTCATCGGTGTCATCATCGACAACTTCAACGAGCAGAAGAAGAAAGCCGGTGGCTCGCTGGAAATG

>GRAA04 Greece Athens(AIA)

CAGGTGGGCAAGCAGCCGATYCGCGAGACCAACATCTACATGTACCTCTACTTYGTGTTCTTCATCATCTKCGGGTCGTTCTTCACCCTCAACCTGTTCATCGGTGTCATCATCGACAACTTCAACGAGCAGAAGAAGAAAGCCGGTGGCTCGCTGGAAATG

>GEBA20 Georgia Batumi

CAGGTGGGCAAGCAGCCGATTCGCGAGACCAACATCTACATGTACCTCTACTTCGTGTTCTTCATCATCTKCGGGTCGTTCTTCACCCTCAACCTGTTCATCGGTGTCATCATCGACAACTTCAACGAGCAGAWGAAGAAWGCCGGTGGCTCKCTGGA----

>PTQT15 Portugal Loul

CAGGTGGGCAAGCAGCCGATTCGCGAGACCAACATCTACATGTACCTCTACTTCGTGTTCTTCATCATCTTCGGGTCGTTCTTCACYCTCAACCTGTTCATCGGTGTCATCATCGACAACTTCAACGAGCAGAAGAAGAAAGCCGGTGGCTCGCTGGAAATG

>ALDU19 Albania Durres

CAGGTGGGCAAGCAGCCGATTCGCGAGACCAACATMTACATGTACCTCTACTTCGTGTTCTTCATCATCTKCGGGTCGTTCTTCACCCTCAACCTGTTCATCGGTGTCATCATCGACAACTTCAACGAGCAGAAGAAGAAAGCCGGTGGCTCGCTGGAAATG

>PTQT14 Portugal Loul

CAGGTGGGCAAGCAGCCAATTCGCGAGACCAACATCTACATGTACCTCTACTTCGTGTTCTTCATCATCTTCGGGTCGTTCTTCACCCTTAATCTGTTCATCGGTGTCATCATCGACAACTTCAACGAGCAGAAGAAGAAAGCCGGTGGCTCGCTGGAAATG

>ESSV16 Spain Sevilla

CAGGTGGGCAASCAGCCGATTCGCGAGACCAACATCTACATGTACCTCTACTTYGTGTTCTTCATCATCTKCGGGTCGTTCTTCACCCTCAACCTGTTCATCGGTGTCATCATCGACAACTTCAACGAGCAGAAGAAGA-----------------------

>ESMO02 Spain Monesterio

CAGGTGGGCAAGCAGCCGATTCGCGAGACCAACATCTACATGTACCTCTACTTCGTGTTCTTCATCATCTTCGGGTCGTTCTTCACYCTCAACCTGTTCATCGGTGTCATCATCGACAACTTCAACGAGCAGAAGAAGAAAGCCGGTGGCTCGCTGGAAATG

>PTPN22 Portugal Penafiel

CAGGTGGGCAAGCAGCCGATTCGCGAGACCAACATCTACATGTACCTCTACTTCGTGTTCTTCATCATCTTCGGGTCGTTCTTCACCCTTAATCTGTTCATCGGTGTCATCATCGACAACTTCAACGAGCAGAAGAAGAAAGCCGGTGGCTCGCTGGAAATG

>PTPN11 Portugal Penafiel

CAGGTGGGCAAGCAGCCAATTCGCGAGACCAACATCTACATGTACCTCTACTTCGTGTTCTTCATCATCTTCGGGTCGTTCTTCACCCTCAATCTGTTCATCGGTGTCATCATCGACAACTTCAACGAGCAGAAGAAGAAAGCCGGTGGCTCGCTGGAAATG

>CRPL14 Croatia Dubrovnik

CAGGTGGGCAAGCAGCCRATTCGCGAGACCAACATCTACATGTACCTCTACTTCGTGTTCTTCATCATCTTCGGGTCGTTCTTCACCCTYAAYCTGTTCATCGGTGTCATCATCGACAACTTCAACGAGCAGAAGAAGAAAGCCGGTGGCTCGCTGGAAATG

>MTLU30 Malta Luqa

CAGGTGGGCAAGCAGCCGATTCGCGAGACCAACATCTACATGTACCTCTACTTYGTGTTCTTCATCATCTTCGGGTCGTTCTTCACCCTCAACCTGTTCATCGGTGTCATCATCGACAACTTCAACGAGCAGAAGAAGAAAGCCGGTGGCTCGCTGGAAATG

>ABSU02 Abkasia Souckhumi

CAGGTGGGCAAGCAGCCGATTCGCGAGACCAACATCTACATGTACCTCTACTTCGTGTTCTTCATCATCTTCGGGTCGTTCTTCACCCTCAACCTGTTCATCGGTGTCATCATCGACAACTTCAACGAGCAGAAGAAGAAAGCCGGTGGCTCGCTGGAAATG

>ITRO09 Italy Roma

-AGGTGGGCAAGCAGCCGATTCGCGAGACCAACATCTACATGTACCTCTACTTCGTGTTCTTCATCATCTTCGGGTCGTTCTTCACCCTCAACCTGTTAATCGGTGTCATCATCGACAACTTCAACGAGCAGAAGAAGAAAGCCGGTGGCTCGCAGGAA---

>CRPL22 Croatia Dubrovnik

CAGGTGGGCAAGCAGCCRATTCGCGAGACCAACATCTACATGTACCTCTACTTCGTGTTCTTCATCATCTTCGGGTCGTTCTTCACCCTYAAYCTGTTCATCGGTGTCATCATCGACAACTTCAACGAGCAGAAGAAGAAAGCCGGTGGCTCGCTGGAAATG

>ITRO07 Italy Roma

CAGGTGGGCAAGCAGCCGATTCGCGAGACCAACATCTACATGTACCTCTACTTCGTGTTCTTCATCATCTTCGGGTCGTTCTTCACCCTCAACCTGTTCATCGGTGTCATCATCGACAACTTCAACGAGCAGAAGAAGAAAGCCGGTGGCTCGCTGGAAATG

>ITRO02 Italy Roma

CAGGTGGGCAAGCAGCCGATTCGCGAGACCAACATCTACATGTACCTCTACTTCGTGTTCTTCAYCATCTTCGGGTCGTTCTTCACCCTYAAYCTGTTCATCGGTGTCATCATCGACAACTTCAACGAGCAGAAGAAGAAAGCCGGTGGCTCGCTGGAAATG

>ITRM08 Italy Roma

---------------CCGATTCGCGAGACCAACATCTACATGTACCTCTACTTCGTGTTCTTCATCATCTSCGGGTCGTTCTTCACCCTCAACCTGTTCATCGGTGTCATCATCGACAACTTCAACGAGCAGAAGAAGAA----------------------

>18_RO_344 Romania Gheorgheni

CAGGTGGGCAAGCAGCCGATTCGCGAGACCAACATCTACATGTACCTCTACTTYGTGTTCTTCATCATCTTCGGGTCGTTCTTCACCCTCAACCTGTTCATCGGTGTCATCATCGACAACTTCAACGAGCAGAAGAAGAAAGCCGGTGGCTCGCTGGAAATG

>ESLU01 Spain Cartagena

CAGGTGGGCAAGCAGCCAATTCGCGAGACCAACATCTACATGTACCTCTACTTCGTGTTCTTCATCATCTTCGGGTCGTTCTTCACCCTCAACCTGTTCATCGGTGTCATCATCGACAACTTCAACGAGCAGAAGAAGAAAGCCGGTGGCTCGCTGGAAATG

>GRCC11 Greece Chania

CAGGTGGGCAAGCAGCCRATTCGCGAGACCAACATCTACATGTACCTCTACTTCGTGTTCTTCATCATCTTCGGGTCGTTCTTCACCCTYAAYCTGTTCATCGGTGTCATCATCGACAACTTCAACGAGCAGAAGAAGAAAGCCGGTGGCTCGCTGGAAATG

>GRAN02 Greece Athens(AIA)

CAGGTGGGCAAGCAGCCGATCCGCGAGACCAACATCTACATGTACCTCTACTTTGTGTTCTTCATCATCTGCGGGTCGTTCTTCACCCTCAACCTGTTCATCGGTGTCATCATCGACAACTTCAACGAGCAGAAGAAGAAAGCCGGTGGCTCGCTGGAAATG

>GRAE09 Greece Athens(AIA)

CAGGTGGGCAAGCAGCCRATTCGCGAGACCAACATCTACATGTACCTCTACTTCGTGTTCTTCAYCATCTTCGGGTCGTTCTTCACCCTTAATCTGTTCATCGGTGTCATCATCGACAACTTCAACGAGCAGAAGAAGAAAGCCGGTGGCTCGCTGGAAATG

>SRAP10 Serbia Apatin

CAGGTGGGCAAGCAGCCRATTCGCGAGACCAACATCTACATGTACCTCTACTTCGTGTTCTTCATCATCTTCGGGTCGTTCTTCACCCTYAAYCTGTTCATCGGTGTCATCATCGACAACTTCAACGARCAGGAGAAGAAAGCCGGTGGCTCGCTGGAAATG

>GRAA03 Greece Athens(AIA)

CAGGTGGGCAAGCAGCCGATTCGCGAGACCAACATCTACATGTACCTCTACTTYGTGTTCTTCATCATCTTCGGGTCGTTCTTCACCCTCAACCTGTTCATCGGTGTCATCATCGACAACTTCAACGAGCAGAAGAAGAAAGCCGGTGGCTCGCTGGAAATG

>GRCN08 Greece Chania

CAGGTGGGCAAGCAGCCAATTCGCGAGACCAACATCTACATGTACCTCTACTTCGTGTTCTTCATCATCTTCGGGTCGTTCTTCACCCTTAATCTGTTCATCGGTGTCATCATCGACAACTTCAACGAGCAGAAGAAGAAAGCCGGTGGCTCGCTGGAAATG

>SLGO30 Slovenia Ajdov ?ina

CAGGTGGGCAAGCAGCCGATTCGCGAGACCAACATCTACATGTACCTCTACTTYGTGTTCTTCATCATCTTCGGGTCGTTCTTCACCCTCAACCTGTTCATCGGTGTCATCATCGACAACTTCAACGAGCAGAAGAAGAAAGCCGGTGGCTCGCTGGAAATG

>GRAN09 Greece Athens(AIA)

CAGGTGGGCAAGCAGCCGATYCGCGAGACCAACATCTACATGTACCTCTACTTTGTGTTCTTCAYCATCTGCGGGTCGTTCTTCACCCTYAAYCTGTTCATCGGTGTCATCATCGACAACTTCAACGAGCAGAAGAAGAAAGCCGGTGGCTCGCTGGAAATG

>GRAN12 Greece Athens(AIA)

CAGGTKGGCAAGCAGCCRATTCGCGAGACCAACATCTACATGTACCTCTACTTYGTGTTCTTCATCATCTTCGGGTCGTTCTTCACCCTYAAYCTGTTCATCGGTGTCATCATCGACAACTTCAACGAGCAGAAGAAGAAAGCCGGTGGCTCGCTGGAAATG

>GRAN05 Greece Athens(AIA)

CAGGTGGGCAAGCAGCCGATTCGCGAGACCAACATCTACATGTACCTCTACTTCGTGTTCTTCATCATCTKCGGGTCGTTCTTCACCCTYAAYCTGTTCATCGGTGTCATCATCGACAACTTCAACGAGCAGAAGAAGAAAGCCGGTGGCTCGCTGGAAATG

>GRAN11 Greece Athens(AIA)

CAGGTGGGCAAGCAGCCGATCCGCGAGACCAACATCTACATGTACCTCTACTTTGTGTTCTTCATCATCTGCGGGTCGTTCTTCACCCTCAACCTGTTCATCGGTGTCATCATCGACAACTTCAACGAGCAGAAGAAGAAAGCCGGTGGCTCGCTGGAAATG

>GRAN01 Greece Athens(AIA)

CAGGTGGGCAAGCAGCCGATCCGCGAGACCAACATCTACATGTACCTCTACTTTGTGTTCTTCATCATCTGCGGGTCGTTCTTCACCCTCAACCTGTTCATCGGTGTCATCATCGACAACTTCAACGAGCAGAAGAAGAAAGCCGGTGGCTCGCTGGAAATG

>GRAN16 Greece Athens(AIA)

CAGGTGGGCAAGCAGCCRATYCGCGAGACCAACATCTACATGTACCTCTACTTYGTGTTCTTCATCATCTKCGGGTCGTTCTTCACCCTYAAYCTGTTCATCGGTGTCATCATCGACAACTTCAACGAGCAGAAGAAGAAAGCCGGTGGCTCGCTGGAAATG

>ESVL14 Spain Catarroja

CAGGTGGGCAAGCAGCCGATTCGCGAGACCAACATCTACATGTACCTCTACTTCGTGTTCTTCATCATCTTCGGGTCGTTCTTCACCCTCAACCTGTTCATCGGTGTCATCATCGACAACTTCAACGAGCAGAAGAAGAAAGCCGGTGGCTCGCTGGAAATG

>GRKA02 Greece Kavala

CAGGTGGGCAAGCAGCCRATYCGCGAGACCAACATCTACATGTACCTCTACTTYGTGTTCTTCATCATCTKCGGGTCGTTCTTCACCCTYAAYCTGTTCATCGGTGTCATCATCGACAACTTCAACGAGCAGAAGAAGAAAGCCGGTGGCTCGCTGGAAATG

>CRPL30 Croatia Dubrovnik

CAGGTGGGCAAGCAGCCGATTCGCGAGACCAACATCTACATGTACCTCTACTTCGTGTTCTTCATCATCTTCGGGTCGTTCTTCACCCTYAAYCTGTTCATCGGTGTCATCATCGACAACTTCAACGAGCAGAAGAAGAAAGCCGGTGGCTCGCTGGAAATG

>GRAE12 Greece Athens(AIA)

CAGGTGGGCAAGCAGCCRATTCGCGAGACCAACATCTACATGTACCTCTACTTYGTGTTCTTCATCATCTTCGGGTCGTTCTTCACCCTYAAYCTGTTCATCGGTGTCATCATCGACAACTTCAACGAGCAGAAGAAGAAAGCCGGTGGCTCGCTGGAAATG

>ESTY09 Spain Torremolinos

CAGGTGGGCAAGCAGCCGATTCGCGAGACCAACATCTACATGTACCTCTACTTCGTGTTCTTCATCATCTTCGGGTCGTTCTTCACCCTCAACCTGTTCATCGGTGTCATCATCGACAACTTCAACGAGCAGAAGAAGAAAGCCGGTGGCTCGCTGGAAATG

>GEBA12 Georgia Batumi

CAGGTGGGCAAGCAGCCGATYCGCGAGACCAACATCTACATGTACCTCTACTTYGTGTTCTTCATCATCTGCGGGTCGTTCTTCACCYTCAACCTGTTCATCGGTGTCATCATCGACAACTTCAACGAGCAGAAGAAGAAAGCCGGTGGCTCGCTGGAAATG

>BG20190001 Bulgaria Burgas

CAGGTGGGCAAGCAGCCGATTCGCGAGACCAACATCTACATGTACCTCTACTTCGTGTTCTTYATCATCTTCGGGTCGTTCTTCACCCTCAACCTGTTSRTCGGTGTCATCATCGACAACTTCAACGAGCAGAAGAAGAAAGCCGGTGGCTCGCTGGAAATG

>19ME390 Montenegro Podgorica

CAGGTGGGCAAGCAGCCGATTCGCGAGACCAACATCTACATGTACCTCTACTTCGTGTTCTTCATCATCTTCGGGTCGTTCTTCACSCTCAACCTGTTCATCGGTGTCATCATCGACAACTTCAACGAGCAGAAGAAGAAAGCCGGTGGCTCGCTGGAAATG

>19ME389 Montenegro Podgorica

CAGGTGGGCAAGCAGCCRATTCGCGAGACCAACATCTACATGTACCTCTACTTCGTGTTCTTCATCATCTTCGGGTCGTTCTTCACCCTYAAYCTGTTCATCGGTGTCATCATCGACAACTTCAACGAGCAGAWGAAGAAAGCCSGTGGCTCGCTGGAAATG

>19ME388 Montenegro Podgorica

CAGGTGGGCAAGCAGCCRATTCGCGAGACCAACATCTACATGTACCTCTACTTCGTGTTCTTCATCATCTTCGGGTCGTTCTTCACCCTCAACCTGTTCATCGGTGTCATCATCGACAACTTCAACGAGCAGAWGAAGAAAGCCGGTGGCTCGCTGGAAATG

>19ME375 Montenegro Podgorica

CAGGTGGGCAAGCAGCCAATTCGCGAGACCAACATCYACATGWACCTSKACTTCGTGTTCTTCATCATCTTCGGGTCGTTCTTCACCCTCAACCTGTTCATYGGTGTCATCATCGACAACTTCAACGAGCAGAAGAAGAAAGCCGGTGGCTCGCWGGAAATG

>ITFI10 Italy Gemona del Friuli

CAGGTGGGCAAGCAGCCGATTCGCGAGACCAACATCTACATGTACCTCTACTTCGTGTTCTTCATCATCTTCGGGTCGTTCTTCACYCTYAAYCTGTTCATCGGTGTCATCATCGACAACTTCAACGAGCAGAAGAAGAAAGCCGGTGGCTCGCTGGAAATG

>ITFI09 Italy Gemona del Friuli

CAGGTGGGCAAGCAGCCGATTCGCGAGACCAACATCTACATGTACCTCTACTTCGTGTTCTTCATCATCTTCGGGTCGTTCTTCACCCTCAACCTGTTCATCGGTGTCATCATCGACAACTTCAACGAGCAGAWGAAGAAAGCCGGTGGCTCGCTGGAAATG

>ITFI08 Italy Gemona del Friuli

CAGGTGGGCAAGCAGCCGATTCGCGAGACCAACATCTACATGTACCTCTACTTCGTGTTCTTCATCATCTTCGGGTCGTTCTTCACCCTYAAYCTGTTCATCGGTGTCATCATCGACAACTTCAACGAGCAGAAGAAGAAAGCCGGTGGCTCGCTGGAAATG

>ITFI07 Italy Gemona del Friuli

CAGGTGGGCAAGCAGCCGATTCGCGAGACCAACATCTACATGTACCTCTACTTCGTGTTCTTCATCATCTTCGGGTCGTTCTTCACCCTCAACCTGTTCATCGGTGTCATCATCGACAACTTCAACGAGCAGAAGAAGAAAGCCGGTGGCTCGCTGGAAATG

>ITBR01 Italy Bari

CAGGTGGGCAAGCAGCCGATTCGCGAGACCAACATCTACATGTACCTCTACTTYGTGTTCTTCAYCATCTTCGGGTCGTTCTTCACSCTYAAYCTGTTCATCGGTGTCATCATCGACAACTTCAACGAGCAGAAGAAGAAAGCCGGTGGCTCGCTGGAAATG

>ITBR16 Italy Bari

CAGGTGGGCAAGCAGCCGATTCGCGAGACCAACATCTACATGTACCTCTACTTCGTGTTCTTCATCATCTTCGGGTCGTTCTTCACCCTCAACCTGTTCATCGGTGTCATCATCGACAACTTCAACGAGCAGAAGAAGAAAGCCGGTGGCTCGCTGGAAATG

>ITBR15 Italy Bari

CAGGTGGGCAAGCAGCCRATTCGCGAGACCAACATCTACATGTACCTCTACTTCGTGTTCTTCATCATCTTCGGGTCGTTCTTCACCCTYAAYCTGTTCATCGGTGTCATCATCGACAACTTCAACGAGCAGAAGAAGAAAGCCGGTGGCTCGCTGGAAATG

>ITBR14 Italy Bari

------------------ATTCGCGAGACCAACATCTACATGTACCTCTACTTCGTGTTCTTCAYCATCTTCGGGTCGTTCTTCACCCTYAAYCTGTTCATCGGTGTCATCATCGACAACTTCAACGAGCAGAAGAAGAAAGCCGGTGGCTCGCTGGAAATG

>GRCN06 Greece Chania

CAGGTGGGCAAGCAGCCRATTCGCGAGACCAACATCTACATGTACCTCTACTTCGTGTTCTTCATCATCTTCGGGTCGTTCTTCACCCTYAAYCTGTTCATCGGTGTCATCATCGACAACTTCAACGAGCAGAAGAAGAAAGCCGGTGGCTCGCTGGAAATG

>TRIS35 Turkey Istanbul

CAGGTGGGCAAGCAGCCGATTCGCGAGACCAACATCTACATGTACCTCTACTTCGTGTTCTTCATCATCTTCGGGTCGTTCTTCACCCTCAACCTGTTCATCGGTGTCATCATCGACAACTTCAACGAGCAGAAGAAGAAAGCCGGTGGCTCGCTGGAAATG

>TRIS33 Turkey Istanbul

CAGGTGGGCAAGCAGCCGATTCGCGAGACCAACATCTACATGTACCTCTACTTCGTGTTCTTCATCATCTTCGGGTCGTTCTTCACCCTCAACCTGTTCATCGGTGTCATCATCGACAACTTCAACGAGCAGAAGAAGAAAGCCGGTGGCTCGCTGGAAATG

>GRAE11 Greece Athens(AIA)

CAGGTGGGCAAGCAGCCGATCCGCGAGACCAACATCTACATGTACCTCTACTTTGTGTTCTTCATCATCTGCGGGTCGTTCTTCACCCTCAACCTGTTCATCGGTGTCATCATCGACAACTTCAACGAGCAGAAGAAGAAAGCCGGTGGCTCGCTGGAAATG

>GRAA10 Greece Athens(AIA)

CAGGTGGGCAAGCAGCCGATYCGCGAGACCAACATCTACATGTACCTCTACTTYGTGTTCTTCATCATCTKCGGGTCGTTCTTCACCCTCAACCTGTTCATCGGTGTCATCATCGACAACTTCAACGAGCAGAAGAAGAAAGCCGGTGGCTCGCTGGAAATG

>ITBR13 Italy Bari

CAGGTGGGCAAGCAGCCGATTCGCGAGACCAACATCTACATGTACCTCTACTTYGTGTTCTTCATCATCTTCGGGTCGTTCTTCACCCTCAACCTGTTCATCGGTGTCATCATCGACAACTTCAACGAGCAGAAGAAGAAAGCCGGTGGCTCGCTGGAAATG

>GEBA40 Georgia Batumi

CAGGTGGGCAAGCAGCCGATCCGCGAGACCAACATCTACATGTACCTCTACTTTGTGTTCTTCATCATCTGCGGGTCGTTCTTCACCCTCAWCCTGTTCATCGGTGTCATCATCGACAACTTCAACGAGCAGAAGAAGAAAGCCGGTGGCTCGCTGGAAATG

>ROBU13 Romania Bucharest

--------------------TCGACAGGTGGGCAAGCAGCCGATTCGCGAGACCAACATCTACATGTACCTCTACTTCGTGTTCTTCATCATCTTCGGGTCGTTCTTCACCCTCAACCTGTTCATGAGG------------------------------------------------------------------------------

>19ME392 Montenegro Podgorica

CGATCGTTTCTCTTGAACCCTTGACAGGTGGGCAAGCAGCCGATTCGCGAGACCAACATCTACATGTACCTCTACTTCGTGTTATTCATCATCTTCGGGTCGTTCTTCACCCTCAACCTGTTCATCGGTGTCATCATCGACAACTTCAACGAGCAGAAGAAGAAAGCCGGTGGCTCGCTGGAAATGTTCATGACGGAGGATCAGAAA

>ESVN04 Spain Madrid

CGATCGTTTCTCTTGAACCCTCGGCAGGTGGGCAAGCAGCCAATTCGCGAGACCAACATCTACATGTACCTCTACTTCGTGTTCTTCATCATCTTCGGGTCGTTCTTCACCCTTAATCTGTTCATCGGTGTCATCATCGACAACTTCAACGAGCAGAAGAAGAAAGCCGGTGGCTCGCTGGAAATGTTCATG---------------

>GRAE03 Greece Athens

CGATCGTTTCCCTTGAATCCTCTACAGGTGGGCAAGCAGCCGATCCGCGAGACCAACATCTACATGTACCTCTACTTTGTGTTCTTCATCATCTGCGGGTCGTTCTTCACCCTCAACCTGTTCATCGGTGTCATCATCGACAACTTCAACGAGCAGAAGAAGAAAGCCGGTGGCTCGCTGGAAATGTTCATGACGGAGGATCAGAAA

>GRAA01 Greece Athens

CGRTCGTTTCTCTTGATYCCTCCACAGGTGGGCAAGCAGCCGATTCGCGAGACCAACATCTACATGTACCTCTACTTYGTGTTCTTCATCATCTTCGGGTCGTTCTTCACCCTCAACCTGTTCATCGGTGTCATCATCGACAACTTCAACGAGCAGAAGAAGAAAGCCGGTGGCTCGCTGGAAATGTTCATGACGGAGGATCAGAAA

>GRCN10 Greece Chania

CGATCGTTTCTCTTGAACCCTCGGCAGGTGGGCAAGCAGCCGATTCGCGAGACCAACATCTACATGTACCTCTACTTCGTGTTCTTCATCATCTTCGGGTCGTTCTTCACCCTCAACCTGTTCATCGGTGTCATCATCGACAACTTCAACGAGCAGAAGAAGAAAGCCGGTGGCTCGCTGGAAATGTTCATGACGGAGGATCAGAAA

>GRCN01 Greece Chania

CGATCGTTTCTCTTGAACCCTCGGCAGGTGGGCAAGCAGCCAATTCGCGAGACCAACATCTACATGTACCTCTACTTCGTGTTCTTCATCATCTTCGGGTCGTTCTTCACCCTTAATCTGTTCATCGGTGTCATCATCGACAACTTCAACGAGCAGAAGAAGAAAGCCGGTGGCTCGCTGGAAATGTTCATGACGGAGGATCAGAAA

>18RS240 Serbia Loznica

CGATCGTTTCTCTTGATCCCTCCACAGGTGGGCAAGCAGCCGATTCGCGAGACCAACATCTACATGTACCTCTACTTTGTGTTCTTCATCATCTTCGGGTCGTTCTTCACCCTCAACCTGTTCATCGGTGTCATCATCGACAACTTCAACGAGCAGAAGAAGAAAGCCGGTGGCTCGCTGGAAATGTTCATGACGGAGGATCAGAAA

>18RS236 Serbia Loznica

CGATCGTTTCTCTTGAWCCCTCSRCAGGTGGGCAAGCAGCCRATTCGCGAGACCAACATCTACATGTACCTCTACTTCGTGTTCTTCATCATCTTCGGGTCGTTCTTCACCCTCAACCTGTTCATCGGTGTCATCATCGACAACTTCAACGAGCAGAAGAAGAAAGCCGGTGGCTCGCTGGAAATGTTCATGACGGAGGATCAGAAA

>TRGN39 Turkey Igneada

CGATCGTTTCTCGTGAATCCTCGACAGGTGGGCAAGCAGCCGATTCGCGAGACCAACATCTACATGTACCTCTACTTCGTGTTCTTCATCATCTTCGGGTCGTTCTTCACTCTCAACCTGTTCATCGGTGTCATCATCGACAACTTCAACGAGCAGAAGAAGAAAGCCGGTGGCTCGCTGGAAATGTTCATGACGGAGGATCAGAAA
